# Biogenic activated carbons from conservation grassland biomass for organic micropollutants removal in municipal wastewater

**DOI:** 10.1016/j.ese.2025.100588

**Published:** 2025-06-06

**Authors:** Korbinian Kaetzl, Marcel Riegel, Ben Joseph, Ronja Ossenbrink, Helmut Gerber, Willis Gwenzi, Tobias Morck, David Laner, Thomas Heinrich, Volker Kromrey, Kevin Friedrich, Michael Wachendorf, Kathrin Stenchly

**Affiliations:** a Section of Grassland Science and Renewable Plant Resources, University of Kassel, Steinstraße 19, 37213 Witzenhausen, Germany; b Section Concepts for Supply Structures and Technology, TZW: DVGW Technologiezentrum Wasser, Karlsruher Straße 84, 76139, Karlsruhe, Germany; c Thünen-Institute of Agricultural Technology, Bundesallee 47, 38116, Brunswick, Germany; d Pyreg GmbH, Trinkbornstraße 15-17, 56281, Dörth, Germany; e Department of Technology Assessment, Leibniz Institute for Agricultural Engineering and Bioeconomy, Max-Eyth-Allee 100, 14469, Potsdam, Germany; f Chair of Urban Water Engineering, University of Kassel, Kurt-Wolters-Straße 3, 34125, Kassel, Germany; g Research Center for Resource Management and Solid Waste Engineering, University of Kassel, Mönchebergtraße 7, 34125, Kassel, Germany; h Department of Postharvest Technologies, Leibniz Institute for Agricultural Engineering and Bioeconomy, Max-Eyth-Allee 100, 14469, Potsdam, Germany; i Bodensee-Stiftung, Fritz-Reichle-Ring 4, 78315, Radolfzell, Germany; j Björnsen Beratende Ingenieure GmbH, Branch Leonberg, Distelfeldstraße 15, 71229, Leonberg, Germany; k Biosystems and Environmental Engineering Research Group, 380 Adylin, Westgate, Harare, Zimbabwe

**Keywords:** Biochar, Life cycle assessment, Pharmaceuticals, Residual biomass, Wastewater treatment

## Abstract

Activated carbons (ACs) are widely used in advanced wastewater treatment to remove organic micropollutants (OMPs), including pharmaceuticals, that evade conventional biological processes. Yet, fossil coal-based ACs generate substantial CO_2_ emissions and conflict with circular-bioeconomy objectives. Here, we address the critical research gap in sustainable sorbent development by evaluating biogenic ACs produced from underutilized grassland biomass. Using a pretreatment to enrich carbon content and reduce minerals, we generated biogenic ACs from wet meadow (WET) and orchard meadow residues and compared them to Norit SAE Super and PULSORB WP 235 in batch adsorption tests. Despite its higher mineral and ash contents and lower specific surface area than conventional ACs, 100 %-activated WET (WET100) combined balanced micro- and mesoporosity—yielding heterogeneous adsorption sites that conform to Freundlich isotherms—and achieved 50 % OMP removal at a dosage of ∼13 mg L^−1^, on par with Norit SAE Super (∼12 mg L^−1^). Strong correlations between OMP removal and ultraviolet absorbance at 254 nm (UVA254; R^2^ > 0.95) validate UVA254 as a rapid monitoring proxy. Greenhouse gas footprint analyses revealed that substituting coal-based AC with WET100 reduces gate-to-grave emissions by approximately 2.4 t CO_2_e per tonne of sorbent—translating to potential savings of up to 94 % CO_2_e when deployed at scale for advanced OMP removal. These findings underscore that biogenic ACs can be seamlessly integrated into existing treatment infrastructure, valorize underutilized grassland biomass, align with circular-economy and EU sustainability objectives, and deliver substantial greenhouse-gas savings compared to coal-based adsorbents.

## Highlights

• Grassland-derived biogenic ACs effectively remove OMPs from municipal wastewater.
• Wet-meadow–derived ACs match fossil ACs in adsorption performance for trace organics.
• Optimized activation enhances mesoporosity and heterogeneity of adsorption sites.
• UVA254 reduction strongly correlates with OMP removal, allowing rapid monitoring.
• LCA reveals up to 17-fold lower CO_2_ emissions for biogenic ACs vs. coal-based ACs.

## Graphical abstract

**Figure:**
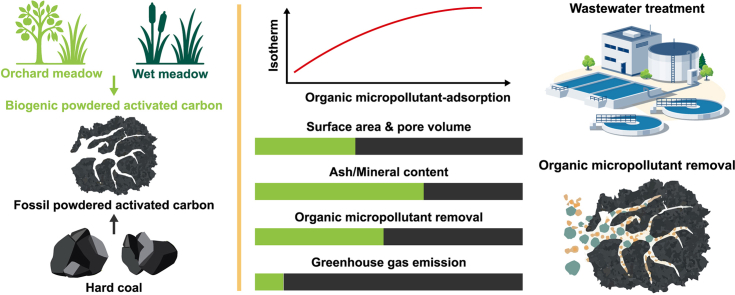

## 1 Introduction

Anthropogenic organic micropollutants (OMPs), including pharmaceuticals, personal care products, and industrial chemicals, pose a severe threat to aquatic ecosystems and the safety of drinking water resources [[1], [2], [3]]. Wastewater treatment plants (WWTPs) represent a major point source of OMPs in surface waters, as many polar OMPs are resistant to conventional biological treatment processes [[4], [5], [6]]. Consequently, advanced treatment strategies are required to mitigate their discharge [4,7]. One of the most effective approaches for OMP removal in WWTPs is adsorption onto powdered activated carbon (PAC) [[8], [9], [10]], typically derived from bituminous coal and coconut shells. However, the widespread use of fossil-based AC contradicts the European Union's vision of a circular bioeconomy based on renewable resources [11]. As a sustainable alternative, biogenic activated carbon derived from residual biomass has gained increasing attention as a sorbent for environmental remediation, including wastewater treatment [12].

Despite promising findings on biogenic ACs produced from lignin-rich feedstocks such as wood residues or fruit stones [[13], [14], [15], [16], [17]], grassland-derived AC has remained largely unexplored. Grassland biomass, particularly from landscape conservation-relevant areas, is frequently underutilized in bioeconomic value chains [18,19] due to its limited suitability for animal feeding [20] and biogas production [21,22]. However, direct conversion of grassland biomass into AC is challenging due to its low lignin content and high mineral concentrations, which can lead to ash-related issues such as slagging, corrosion, and emissions during pyrolysis [[23], [24], [25], [26]].

To improve the suitability of grassland biomass for AC production, the Integrated Solid Fuel and Biogas Production from Biomass (IFBB) process has been proposed as a pre-treatment technique [12,27,28]. This process separates biomass into two fractions: a press fluid rich in soluble organic matter, suitable for biogas production, and a press cake with enhanced carbon content and reduced mineral load, which may serve as a more suitable precursor for AC production [12,29].

**Fig. 1:**
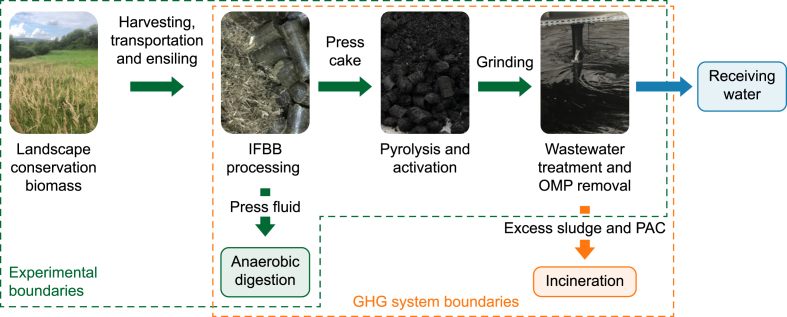
Schematic representation of the process chain converting landscape conservation biomass into activated carbon for advanced wastewater treatment. Experimental boundaries (green dashed line) include biomass harvesting, Integrated Generation of Solid Fuel and Biogas from Biomass (IFBB) processing, pyrolysis, activation, and application for organic micropollutant (OMP) removal. System boundaries for greenhouse gas (GHG) assessment (orange dashed line) cover associated mass and energy flows, including press fluids and excess sludge containing powdered activated carbon (PAC).

This study evaluates the feasibility of grassland-derived AC as an alternative adsorbent for removing OMPs in wastewater treatment applications. To this end, the adsorption performance of grassland-based AC is systematically compared with conventional fossil-based ACs. The experimental approach includes material characterization to evaluate the physicochemical properties of the produced ACs, including specific surface area, pore structure, and elemental composition. Adsorption experiments are conducted using selected OMPs, such as pharmaceuticals (irbesartan, diclofenac), corrosion inhibitors (benzotriazole), and X-ray contrast agents (iohexol, iopamidol), to assess removal efficiencies at varying AC dosages. Isotherm modelling elucidates the adsorption mechanisms and provides insights into the interactions between AC and OMPs. Finally, an environmental impact assessment is undertaken, focusing on the greenhouse gas (GHG) footprints of the generated ACs, to contextualise their sustainability benefits compared to conventional AC products (Fig. 1).

## 2 Materials and methods

### 2.1 Biomass processing and characterization

Two different landscape conservation materials (LCM) were used as feedstocks for AC production. The undergrowth of an orchard meadow (ORC) and a wet meadow (WET) from the Lake Constance District (Southern Germany) were harvested in compliance with nature conservation schemes in September and preserved as silage for further processing by the IFBB technology as described in detail by Joseph et al. [30]. Silage was shredded and mashed in warm water (40 °C) for 15 min. After that, mashed silage was mechanically dewatered by a screw press (type AV, Anhydro Ltd., Kassel, Germany) and separated in a solid press cake (PC) and press fluid. The PC was dried and pressed into 6 mm pellets (qteck PP230UG, qteck GmbH, Bergen, Germany) for AC production. Samples of raw materials (silage), PC, and press fluid were taken for ultimate, proximate, and fibre analyses. Water content was determined by drying at 105 °C to a constant mass (DIN 51718). Ash content was determined on dry matter (DM) by heating to constant mass at 550 °C (DIN 51719). Carbon (C), hydrogen (H), and nitrogen (N) were determined according to the standard method (DIN 51732) using a vario MAX CHN elemental analyser. Sulphur (S) was determined with a CHS-500 analyser (ELTRA GmbH, Germany) according to DIN 16948/16994. Oxygen (O) content was calculated by mass balance according to DIN 51733. Higher heating values (HHV) and lower heating values (LHV; Supplementary Table S1) were calculated according to Bychkov et al. [31]. Fibre analysis (NDF, ADF, and ADL) was conducted using ANKOM 200 Fibre Analyser (ANKOM technology, United States). Main elements were measured with inductively coupled plasma–optical emission spectrometry (ICP–OES; SPECTROBLUE, Spectro Analytical Instruments, Kleve, Germany) after microwave digestion with concentrated nitric acid. All analyses were conducted in triplicate.

The biomethane potential (BMP) of the IFBB-generated press fluid was determined in triplicate using a 20-Litre batch system as described in detail by Richter et al. [22] in accordance with the German Standard VDI 4630 [32]. The BMP was calculated based on the volatile solid (VS) content of the press fluid.

### 2.2 Biochar and AC production

IFBB press cake pellets were converted into AC using a continuously operated laboratory auger reactor (PYREKA; PYREG GmbH, Doerth, Germany; Supplementary Fig. S1). ACs were produced in a one-step pyrolysis and physical activation process at 900 °C with a residence time of 25 min (Supplementary Table S2), conditions that were selected based on previous experiments with grass biomass, which demonstrated that these parameters yield an optimal balance between carbonization efficiency and desired AC properties. Steam (H_2_O) was used as an activation agent and was applied in three quantities to the pyrolysis to produce three ACs from ORC and WET. The oxidation level was defined as the stoichiometric share of oxygen, which was added by steam to oxidize 0 % (ORC0 and WET0), 50 % (ORC50 and WET50), or 100 % (ORC100 and WET100) of the biomass carbon content. The simplified partial oxidation reaction during the pyrolysis and activation at 900 °C was assumed to be:

(1) $$C\left(s\right)+H_{2}O\left(g\right)\;\to \;\text{CO}\left(g\right)+H_{2}\left(g\right)$$

where C(s) indicates the carbon component of the biomass, H_2_O(g) signifies water vapour in the gaseous phase, while CO(g) and H_2_(g) represents the resulting oxidation products in the pyrolysis gas. To ensure steady state conditions, carbonates produced during the reactor's run-in period (first 50 min) and run-out period were discarded. AC was produced for at least 60 min to ensure representative production and reliable conversion data. Approximately 5 kg of biomass pellets were used for each AC batch, and aliquots for analysis were taken in accordance with DIN EN ISO 18135.

The three ACs derived from each biomass were analysed alongside two commercial PACs, NORIT® SAE Super (Norit Activated Carbon B.V., the Netherlands; SAE) and PULSORB® WP 235 (Chemviron Carbon, Belgium; WP 235), which serve as industry benchmarks for WW treatment.

### 2.3 AC characterization

All carbon products were ground using a vibration mill to an average particle size of less than 50 μm (Retsch MM 500, Retsch, Germany). The particle size was confirmed by laser diffraction (Partica LA-960, HORIBA, Ltd., Japan). The PACs were characterised regarding C, H, and N according to DIN 51732 using a vario MAX CHN elemental analyser (Elementar Analysensysteme, Germany). The S content was determined with a CHS-500 analyser (ELTRA, Germany) according to DIN 16948/16994. The O content was calculated by mass balance according to DIN 51733. Water and ash content were determined according to DIN EN 12902 at 150 °C and 650 °C, respectively. The specific surface area (SSA) and pore size distribution (PSD) were determined by nitrogen (N_2_) adsorption and desorption at 77.35 K using the 3P Sync440A (3P Instruments GmbH & Co. KG, Odelzhausen, Germany) gas adsorption analyser. SSA was calculated via the Brunner–Emmett–Teller (BET) method (DIN ISO 9277:2014–01) within a relative pressure range of 0.005–0.1. The micropore area was evaluated using the t-plot method (DIN 66135–1:2001–06), while the macro- and mesopore area was obtained as the difference between the SSA and the micropore area. The distribution of mesopores was further characterised by the Barrett–Joyner–Halenda (BJH) method [33]. The average pore diameter (4V/A) was calculated under the assumption of a cylindrical pore geometry. The iodine and nitrobenzene number, determined in accordance with DIN EN 12902 and DVGW (2011) [34], served as proxies to evaluate the adsorption capacity of ACs, indirectly reflecting their porosity and available surface area for small and aromatic molecules.

The main elements were determined with ICP–OES (SPECTROBLUE, Spectro Analytical Instruments, Kleve, Germany) after microwave digestion with concentrated nitric acid. Electrical conductivity (EC) and pH of ACs were measured according to Singh et al. [35]. Briefly, 5 g of dried PAC was dissolved in 50 mL deionised water and shaken on a horizontal lab shaker for 1 h. After a sedimentation time of 30 min, the EC and pH of the suspension were measured. All analyses, besides SSA and PSD, were conducted in triplicate. Zeta potential was measured once per sample using a Malvern ZSU 5700 zetasizer with DTS 1070 cuvettes at 25 °C. A 1 mg sample of PAC was suspended in 1 mL deionised water, and the pH was adjusted to ∼8.1 by adding HCl to match that of WW. All samples were ultrasonicated before measurement.

### 2.4 WWTP effluent samples and OMP selection

Effluent from the secondary clarifier of the municipal wastewater treatment plant (WWTP) in Kressbronn-Langenargen, Baden-Württemberg, Germany—serving approximately 25,600 population equivalents—was collected for adsorption batch experiments. The effluent was characterised for chemical oxygen demand (COD), total solids (TS), and total suspended solids (TSS). COD was determined using cuvette tests LCK1414 (Hach, United States), while TS and TSS were analysed according to DIN 38409 standards. The measured values were 43.5 mg L^−1^ for TS, 5.8 mg L^−1^ for TSS, and 19.1 mg L^−1^ for COD (Supplementary Table S3). WW was analysed for the concentrations of 15 OMPs that were defined as the so-called KomS-B list by the Micropollutants Competence Centre Baden-Württemberg, Germany (KomS-BW) for operations monitoring of WWTPs [36]. For adsorption analyses and evaluation of AC performance, the KomS-B list was divided into three OMP groups (Table 1). OMP Group 1 includes seven trace substances and commonly serves as an indicator group for monitoring adequate trace substance elimination performance by 80 % in the complete WWTP [5,36]. OMP Group 2 included all OMPs from Group 1 and was supplemented with the three pharmaceuticals ibuprofen, candesartan, and sulfamethoxazole. OMP Group 3 comprises all OMPs investigated, including X-ray contrast agents.

**Table 1:** Initial concentrations (*C*_0_) of organic micropollutants (OMPs) from three substance groups in the effluent of a wastewater treatment plant in Kressbronn–Langenargen, Germany, alongside their log*D* values at pH 8 and molecular weights.

Substance	Substance group	ID	*C*_0_ (μg L^−1^)	log*D* (at pH 8)	Molecular weight (g mol^−1^)
**OMP Group 1 (on average 80 % removal efficiency of six samples)**					
Ʃ4(5)-Methylbenzotriazole	Corrosion inhibitors	MeBT	1.05	1.7	133.15
Benzotriazole	Corrosion inhibitors	BTA	4.30	1.3	119.12
Irbesartan	Pharmaceuticals	IBS	1.25	2.9	428.94
Carbamazepine	Pharmaceuticals	CBZ	2.15	2.5	236.27
Diclofenac	Pharmaceuticals	DFN	1.95	0.9	296.15
Metoprolol	Pharmaceuticals	MTP	2.20	0.2	267.36
Hydrochlorothiazide	Pharmaceuticals	HCT	2.00	−0.3	297.70

**OMP Group 2 (= OMP Group 1 + following substances)**					
Ibuprofen	Pharmaceuticals	IBU	2.00	0.3	206.28
Candesartan	Pharmaceuticals	CDS	2.55	−0.6	440.44
Sulfamethoxazole	Pharmaceuticals	SMX	1.65	−0.9	253.28

**KomS-B X-ray contrast agents list (optional expansion of KomS-B list; included in OMP Group 3)**					
Amidotrizoic acid	X-ray contrast agents	ATA	2.45	−1.3	613.91
Iopromide	X-ray contrast agents	IoPR	1.55	−1.3	791.12
Iomeprol	X-ray contrast agents	IoMP	0.82	−2.8	777.12
Iopamidol	X-ray contrast agents	IoPA	1.85	−1.5	777.09
Iohexol	X-ray contrast agents	IoHX	2.05	−2.6	821.14

Since the background concentration of the investigated OMPs in the WW was partly very low and close to or below the limit of quantification (LOQ; Supplementary Table S3), specific OMP concentrations were adjusted to the normal range by adding purified substances (Table 1). This ensured not only an initial concentration (*C*_0_) of OMPs above the LOQ, but also sufficient equilibrium OMP concentrations (*C*_e_) in adsorption experiments to calculate the corresponding isotherms. To clarify the OMP removal performance of the tested ACs, OMPs’ log*D* and molecular weight were used. The log*D* value is related to the octanol-water partition coefficient (*K*_OW_) and serves as a measure of the lipophilicity (fat solubility) or hydrophilicity (water solubility) of a substance. log*D* is positive for lipophilic (apolar) and negative for hydrophilic (polar) substances [5].

### 2.5 Adsorption experiments with WWTP effluent

The adsorption capacities of all ACs were determined by performing batch experiments on PAC stock suspensions (2 g L^−1^ in demineralized water), according to Zietzschmann et al. [37]. Briefly, four determined PAC concentrations, namely 5, 10, 20, and 40 mg L^−1^, were obtained by pipetting the appropriate volume of stock solution to 200 mL WW in 300 mL flasks and shaking on a horizontal lab shaker at 180 rpm for 24 h. According to Zhang et al. [38] and Zietschmann et al. [39] as well as our findings (Supplementary Fig. S2), a contact time of 24 h is sufficient to achieve equilibrium conditions. The maximum dilution of WW was 2 % (at 40 mg L^−1^). Afterwards, PAC was removed from the suspension using 0.45 μm membrane syringe filters (ROTILABO®, Carl Roth GmbH + Co. KG, Germany). The first 2–3 mL of the filtrate were discarded, and the rest was collected for spectrometry analyses (UVA_254_) and OMP quantification. A zero sample of 200 mL of WWTP effluent was treated equally in all steps with three replicates.

Ultraviolet (UV) adsorption was measured in 5 cm quartz test cells at 254 nm using a DR 6000 photometer (Hach-Lange, Germany). The OMP concentrations were analysed via high-performance liquid chromatography tandem mass spectrometry (HPLC–MS/MS) according to DIN 38407-47 with a 1290 Infinity II (Agilent) and MS/MS QTRAP 6500+ (AB SCIEX, USA) with a LOQ of 0.1 μg L^−1^.

### 2.6 Isotherm calculation and statistics

The OMP removal was calculated based on the results of the adsorption tests. For values below the LOQ, the detection limit with 0.1 μg L^−1^ was used in the calculation. A rational equation (equation (2)) was used to describe the removal efficiency of OMP and UVA_254_ as a function of the PAC dosage, as well as the OMP removal efficiency as a function of UVA_254_ reduction.

(2) $$y\left(x\right)=\frac{ax}{1+bx}$$

Here, y represents the removal efficiency of either OMP or UVA_254_, and *x* is the independent variable PAC dosage for removal as a function of PAC, or UVA_254_ reduction for OMP removal as a function of UVA_254_ reduction. The constant *a* modulates the adsorption rate and establishes the initial adsorption efficiency, while constant *b* regulates the saturation of the adsorption process. Based on the results of the adsorption tests, adsorption isotherms were calculated using the Langmuir (equation (3)) [40] and the Freundlich (equation (4)) [41] equations. Isotherms were calculated for all OMPs and ACs, as well as for each of the three OMP groups. The groups' OMP concentration was calculated by combining the concentrations of the OMPs in each group.

(3) $$Q_{e}=\frac{K_{L}\times C_{e}\times q_{\max}}{1+K_{L}\times C_{e}}$$

in the Langmuir adsorption isotherm (equation (3)), *Q*_e_ (μg OMP per mg AC) is the equilibrium adsorption capacity, *C*_e_ (μg OMP L^−1^) is the equilibrium concentration of the OMP in solution, *q*_max_ (μg OMP per mg AC) is the maximum adsorption capacity of AC, and *K*_L_ (L mg^−1^) is the Langmuir constant.

(4) $$Q_{e}=K_{F}{C_{e}}^{\frac{1}{n}}$$

in the Freundlich adsorption isotherm (equation (4)), *Q*_e_ (μg OMP per mg AC) is the equilibrium adsorption capacity, *C*_e_ (μg OMP L^−1^) is the equilibrium concentration of OMP in solution, *K*_F_ (L^1/*n*^ mg^(1−1/*n*)^ g^−1^) is the Freundlich constant indicative of adsorption capacity, and *n* is the heterogeneity factor related to adsorption intensity. The values of *K*_F_ and *n*, along with the adjusted *R*^2^ as the coefficient of determination, were calculated for each OMP and the three determined substance groups (Table 1).

Based on the calculated Freundlich coefficients, the required quantities of PAC (*M*; mg AC L^−1^) needed to reduce the combined OMP concentration of the three groups by 50 % were determined using equation (5) [42]:

(5) $$M=\frac{V\times R\times C_{0}^{\left(1-\frac{1}{n}\right)}}{K_{F}\times {\left(1-R\right)}^{\frac{1}{n}}}$$

where *V* (L) is the volume of the solution, *R* is the target reduction of OMP, *C*_0_ (μg OMP L^−1^) is the initial OMP concentration in the solution, *K*_F_ (L^1/*n*^ mg^(1−1/*n*)^ g^−1^) is the Freundlich constant indicative of adsorption capacity, and *n* is the heterogeneity factor related to adsorption intensity. To prevent theoretical PAC dose levels over the measured range of 40 mg L^−1^, a 50 % elimination was specified. A 50 % reduction was applied instead of the conventional 80 % [5,36]. This approach assumes (1) partial biological degradation of OMPs in the WWTP [43], (2) additional adsorption of external PAC recirculation, and (3) further degradation of post-filtration. Additionally, it accounts for the fact that initial OMP concentrations were artificially elevated, thereby exceeding the typical range (Supplementary Table S3).

Calculating and plotting isotherm models was conducted in R version 4.3.2 [44], with additional functions provided by the package SorptionAnalysis [45].

### 2.7 Greenhouse gas footprint of generated AC products

In a ‘gate to grave’ approach in which ‘gate’ refers to the entry point of raw material to the WWTP and ‘grave’ alludes to the incineration of the PACs as the final process, the GHG footprint (expressed as global warming potential over 100 years, GWP_100_) of the generated AC products from LCMs was calculated and compared to the two conventionally used ACs, SAE Super and WP 235. Life cycle inventory (quantification of material and energy inputs and outputs; Supplementary Fig. S3 and S4) as well as system boundaries (Fig. 1) were set as detailed by Joseph et al. [12]. We set the functional unit (FU) to the amount (in metric tonnes) of AC needed to remove 50 % of OMP from 1000 m^3^ of WW. The greenhouse gas footprint was only calculated for the best-performing biogenic ACs from both LCMs, ORC100 and WET100, and compared to the conventional ACs. The GHG footprint analyses have been performed using the software tool openLCA 1.9.0 [46].

## 3 Results

### 3.1 Mass flow, pyrolysis feedstock characteristics, and press fluids’ methane yield

The two investigated LCMs showed a notable difference in their physico-chemical composition. With nearly 20 % DM, the ash content of the ORC silage was almost four times higher than in the WET silage (5.4 % DM; Table 2). Besides higher metal and earth alkaline metal content (e.g., Fe, Al, and Ca), ORC silage was also characterised by higher hemicellulose (207 g kg^−1^, WET: 175 g kg^−1^) and lower cellulose (247 g kg^−1^; WET: 295 g kg^−1^) content (Supplementary Table S1).

**Table 2:** Physico-chemical properties (mean values, all on dry matter basis) of press cake (PC) and silage (in parentheses) from orchard meadow (ORC) and wet meadow (WET), used as feedstock for activated carbon (AC) production at 900 °C with 25 min residence time and 0 %, 50 % and 100 % oxidation, compared to coal-based ACs Norit SAE Super (SAE) and Pulsorb WP 235.

Parameter	Unit	Feedstock		Biogenic ACs						Standard ACs	
		ORC	WET	Orchard meadow (ORC)			Wet meadow (WET)			SAE	WP 235
		PC (Silage)	PC (Silage)	0 %	50 %	100 %	0 %	50 %	100 %		
Ash	%	9.6 (19.4)	3.2 (5.4)	37.3	43.8	48.3	13.0	15.3	18.9	9.5	13.4
C	%	45.9 (40.7)	49.4 (48.0)	46.5	43.2	40.5	69.3	66.1	59.2	82.8	79.3
O	%	37.9 (33.7)	40.8 (39.8)	5.3	4.1	5.4	5.2	6.1	6.9	3.8	3.6
H	%	5.4 (4.8)	5.6 (5.5)	1.2	0.9	0.8	1.2	1.3	1.7	0.1	0.1
N	%	1.3 (1.4)	1.0 (1.1)	0.7	0.6	0.6	1.0	0.7	0.7	0.3	0.3
S	%	0.08 (0.09)	0.03 (0.18)	0.18	0.09	0.07	0.20	0.15	0.12	0.60	0.67
O/C ratio	–	0.83	0.83	0.07	0.06	0.09	0.05	0.06	0.07	0.03	0.03
H/C ratio	–	0.12	0.11	0.26	0.22	0.21	0.17	0.21	0.28	0.01	0.01
Ca	g per kg	11.3 (10.7)	7.9 (8.4)	47.7	52.6	57.8	37.2	51.5	41.4	3.5	2.9
Fe	g per kg	2.8 (3.8)	0.1 (0.2)	8.2	8.5	20.2	2.3	4.2	5.1	1.0	0.7
Mg	g per kg	1.5 (1.2)	0.3 (3.7)	7.9	8.7	9.5	10.1	13.5	13.5	2.4	1.7
Al	g per kg	3.1 (2.7)	0.07 (0.2)	9.4	10.7	11.8	0.5	0.7	0.6	11.6	9.3
Na	g per kg	1.1 (1.6)	0.4 (1.0)	1.4	1.6	1.6	1.2	1.5	1.5	1.0	0.7
P	g per kg	1.1 (1.4)	0.3 (0.8)	3.7	4.0	4.4	2.0	2.7	2.7	0.7	0.5
Cu	g per kg	0.01 (0.01)	0.01 (0.01)	0.06	0.07	0.09	0.05	0.07	0.07	0.01	0.02
Cr	g per kg	0.05 (0.04)	0.003 (0.002)	0.19	0.07	1.95	0.11	0.32	0.48	0.04	0.04
K	g per kg	1.58 (10.4)	1.35 (8.0)	8.3	8.7	10.1	7.1	9.4	9.5	0.5	0.3
Ni	g per kg	0.12 (0.21)	0.02 (0.02)	0.36	0.03	0.12	0.02	0.04	0.03	0.04	0.06
Mn	g per kg	0.23 (0.25)	0.03 (0.05)	0.79	0.84	1.04	0.18	0.24	0.25	0.19	0.20
Zn	g per kg	0.04 (0.04)	0.04 (0.04)	0.10	0.11	0.16	0.08	0.13	0.16	0.04	0.01
BET specific surface area	m^2^ g^−1^	–	–	218	272	262	347	460	437	1100	1090
Micropore volume (<2 nm)	cm^3^ g^−1^	–	–	0.07	0.10	0.10	0.13	0.14	0.15	0.34	0.30
Mesopore volume (2–50 nm)	cm^3^ g^−1^	–	–	0.10	0.17	0.16	0.08	0.20	0.23	0.33	0.35
Total pore volume	cm^3^ g^−1^	–	–	0.17	0.27	0.26	0.21	0.34	0.38	0.67	0.65
Iodine number	mg g^−1^	–	–	209	331	278	320	559	559	1044	1173
Nitrobenzene number	mg L^−1^	–	–	122.5	60.4	62.0	37.7	30.4	31.5	23	22.2
pH	–	–	–	10.3	11.4	11.3	12.1	11.3	11.8	9.7	9.3
Electrical conductivity (EC)	μS cm^−1^	–	–	766	611	633	2460	1626	2100	169	146
Zeta potential	mV	–	–	−16.7	−11.5	−12.7	−15.9	−11.5	−12.0	−12.4	−17.7

Compared to their raw material (silage), the IFBB press cake of both LCMs, which served as feedstock for AC production, contained less ash and minerals, while C and O were concentrated (Table 2). This was accompanied by a higher LHV of both produced PCs compared to the silage, namely from 15.6 to 17.2 MJ kg^−1^ for ORC and from 18.0 to 18.5 MJ kg^−1^ for WET, while HHV was increased from 16.5 to 18.2 for ORC and from 19.0 to 19.6 MJ kg^−1^ for WET (Supplementary Table S1). Potentially harmful elements for combustion, such as Cl and S, were reduced in the generated PC, with a significant proportion of Cl transferred into the press fluid. This reduced from 0.10 % DM to 0.05 % DM (ORC) and from 0.68 % DM to as low as 0.02 % DM (WET).

Comparing the PC quality of the two LCMs, a significantly higher ash content in ORC than WET was found. The PC of ORC was further characterised by slightly lower contents of C, H, and O and higher content of Fe and Al. Overall, the fibre content in the PC was increased by the IFBB process. Although the lignin content (ADL) of both LCM press cakes was similar at around 100 g per kg DM, the hemicellulose content was higher in ORC PC (245 g per kg DM) than in WET PC (201 g per kg DM), while the cellulose content was with 321 g per kg DM lower in ORC PC compared to 362 g per kg DM in WET PC. The respective BMPs from the anaerobic digestion of the press fluids, extracted through the IFBB procedure, were 102 standard litres (NL) per kg VS (ORC) and 129 NL per kg VS (WET).

### 3.2 Conversion and physico-chemical properties of ACs

The conversion rate of biomass to AC differed significantly between the two LCMs. In general, the conversion rate decreased with increasing oxidation potential. Consequently, the yield of biogenic AC from ORC was 22.6 % DM (0 % activation), 19.1 % DM (50 %) and 17.2 % DM (100 %) and as such, higher than for WET with 16.9 % DM (0 %), 14.3 (50 %) and 11.7 % DM (100 %; Supplementary Table S2).

The primary chemical components affected by oxidation were ash, which increased, and carbon, which decreased, with higher oxidation levels (Table 2). The ash and C content of the biogenic ACs varied significantly. Unlike AC derived from WET, which contained up to 19 % DM of ash and at least 59 % DM of C (WET100), AC made from ORC (ORC100) contained substantially more ash (up to 48 %) and less carbon (40 % DM). ORC-based ACs also showed notably higher Fe and Al levels.

Regarding pH, ACs from WET biomass exhibited slightly higher values (11.3–12.1) than ORC-derived ACs (10.3–11.3). Commercial ACs had the lowest pH, with less than 10. A striking difference was evident in EC: WET ACs reached up to 2460 μS cm^−1^, more than ten times higher than reference ACs and more than twice that of ORC ACs.

With rising oxidation potential, SSA and pore volume clearly increased (Table 2). Both parameters were higher in AC derived from WET than from ORC, reaching maximum values at 100 % oxidation potential. The iodine number, used as a proxy for SSA, correlated positively with SSA. Adsorption capacity, indicated by the nitrobenzene number, varied significantly: ORC100 reached 62 mg L^−1^, while WET100 (30 mg L^−1^) was comparable to conventional ACs (23 mg L^−1^).

The reference ACs, Norit SAE Super and Pulsorb WP 235, showed only minor differences in chemical and physical properties. Both were characterised by low ash content, high carbon (∼80 % DM) and sulphur content (∼0.6 % DM), an SSA exceeding 1100 m^2^ g^−1^, and a total pore volume of ∼0.65 cm^3^ g^−1^. Consequently, SSA, total pore volume, and mesoporosity in the reference ACs were approximately twice as high, or even greater, than in their biogenic counterparts (Table 2).

### 3.3 Adsorptive OMP removal by AC products

**Fig. 2:**
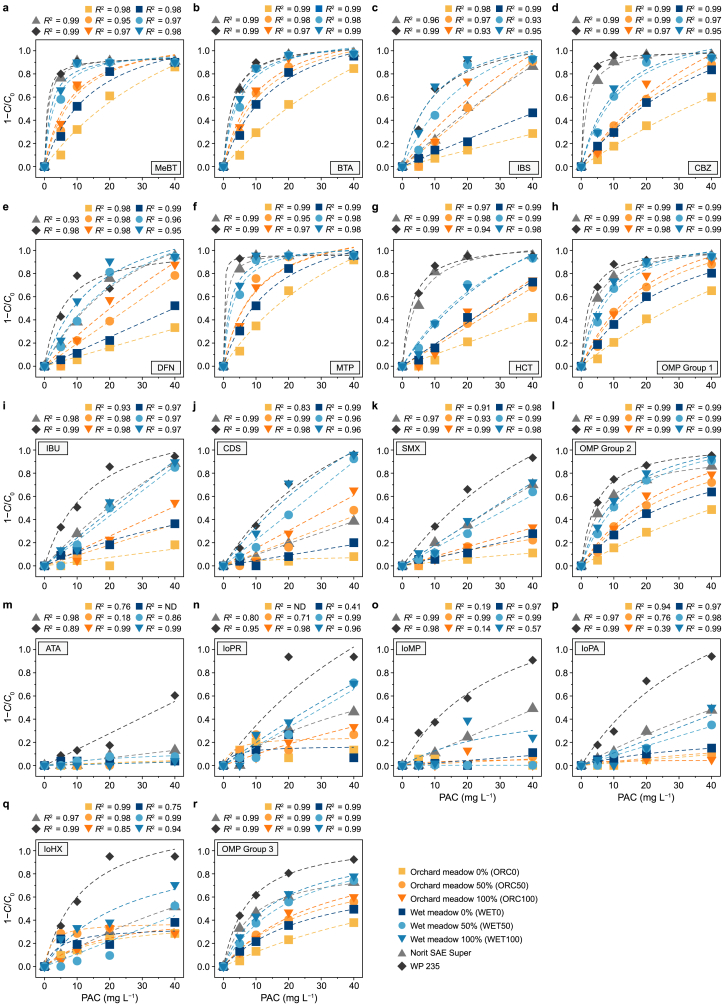
Normalized organic micropollutant (OMP) reduction (1−*C*/*C*_0_) as a function of powdered activated carbon (PAC) dosage after 24 h equilibration. The initial OMP concentration *C*_0_ was determined in triplicate, and the equilibrium concentration *C* at different PAC dosage was obtained from single measurements. Data were fitted with $y\left(x\right)=\frac{ax}{1+bx}$, where *y* is the OMP removal efficiency and *x* is the PAC dosage. The corresponding coefficient of determination (*R*^2^) is shown above each subplot. ND signifies that the equation for specific PAC and OMP was not defined. **a**–**h**, OMP Group 1: ≥80 % wastewater treatment plant removal of MeBT (**a**), BTA (**b**), IBS (**c**), CBZ (**d**), DFN (**e**), MTP (**f**), and HCT (g); cumulative removal across all Group 1 compounds is shown in panel **h**. **i**–**l**, OMP Group 2: IBU (**i**), CDS (**j**), and SMX (**k**); cumulative removal of OMP Groups 1 and 2 is presented in panel **l**. **m**–**r**, X-ray contrast agents: ATA (**m**), loPR (**n**), IoMP (**o**), loPA (**p**), and loHX (**q**); total cumulative removal of all monitored OMPs is shown in panel **r**. Legend specifies AC type and oxidation level (50 % or 100 %) from orchard meadow (ORC) and wet meadow (WET), versus Norit SAE Super and WP 235.

Overall, the adsorption performance of all tested ACs was strongly influenced by the physico-chemical properties of the OMPs, with removal efficiency tending to decline as molecular weight increased and log*D* values decreased. The normalized removal (1−*C*/*C*_0_) of OMPs differed significantly across the studied OMPs and ACs, making a comparison difficult (Fig. 2a–g, i–k, and m–q). To facilitate comparison, OMPs were grouped into three categories (OMP Groups 1, 2, and 3), and isotherms were modelled accordingly (Supplementary Fig. S5).

**Fig. 3:**
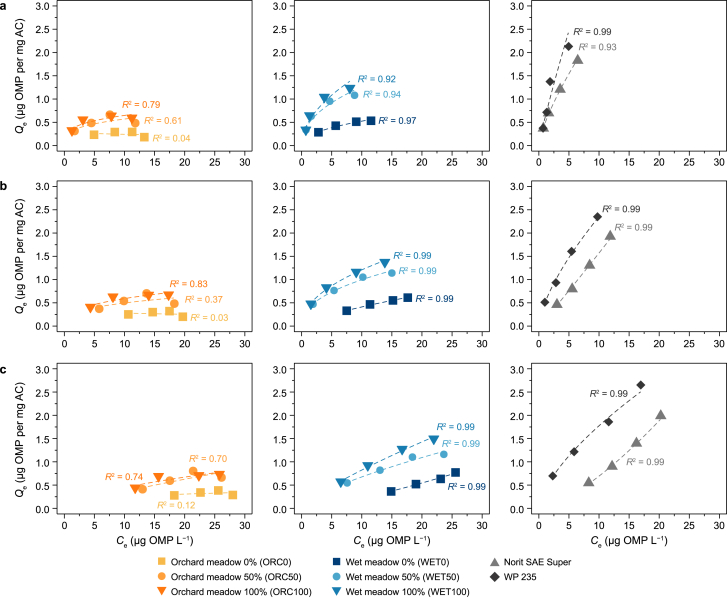
Freundlich isotherms for organic micropollutant (OMP) sums across three clusters: biogenic activated carbons (ACs) from orchard meadow (left), wet meadow (middle), and reference ACs (right). **a**, OMP Group 1: OMPs requiring ≥80 % removal in wastewater treatment plants (MeBT, BTA, IBS, CBZ, DFN, MTP, and HCT). **b**, OMP Group 2: Group 1 plus IBU, CDS, and SMX. **c**, OMP Group 3: 15 OMPs from Koms-B list (Groups 1 and 2, plus ATA, IoHX, IoMP, IoPA, and IoPR). *Q*_e_: the equilibrium AC loading after 24 h; *C*_e_: the corresponding OMP equilibrium concentration in solution; *R*^2^: the Freundlich isotherm's coefficient of determination. Dashed lines represent fitted Freundlich isotherms.

OMP Group 1 comprised compounds targeted for ≥80 % removal. Across the tested biogenic ACs, those derived from WET biomass demonstrated markedly better performance than those from ORC biomass. This trend was particularly evident when comparing non-oxidised and fully oxidised carbons (Fig. 3). For instance, WET0 achieved a *K*_F_ of 0.19 with an *R*^2^ of 0.97, whereas ORC0 recorded a *K*_F_ of 0.31 but with a much lower *R*^2^ of 0.04 (Table 3). With increasing oxidation levels, both *K*_F_ and *R*^2^ generally improved. WET100 showed a *K*_F_ of 0.47 (*R*^2^ = 0.92), almost matching SAE at 0.47 (*R*^2^ = 0.99), albeit still below WP 235, which reached 0.58 (*R*^2^ = 0.93). A key practical parameter is the required PAC dosage to achieve a 50 % reduction in Group 1 OMPs. With WET100 5.5 mg L^−1^ was needed, substantially less than 12.1 mg L^−1^ for ORC100. However, WP 235 and SAE both required less PAC (2.1 mg L^−1^ and 3.6 mg L^−1^, respectively). This pattern also held across OMP Group 2, representing moderately regulated contaminants. WET-based carbons surpassed ORC-based carbons, and higher oxidation levels yielded better adsorption. PAC demand followed a similar trend: 8.4 mg L^−1^ was needed for WET100 to remove 50 %, whereas with ORC100 17.3 mg L^−1^ was required. Among the commercial references, WP 235 stood out with a *K*_F_ of 0.50 and near-complete model fit (*R*^2^ = 0.99).

**Table 3:** Freundlich isotherm coefficients (*K*_F_, *n*, *p*, *R*^2^) of the three organic micropollutant (OMP) groups across biogenic activated carbons (ACs) from orchard meadow and wet meadow at 0 %, 50 %, and 100 % activation, compared to coal-based ACs SAE Supera and WP 235. PAC demand (mg L^−1^) for 50 % OMP removal and substitution factor relative to SAE Super are provided.

OMP Group	Coefficient	Biogenic Acs						Standard ACs	
		Orchard meadow (ORC)			Wet meadow (WET)			SAE Super	WP 235
		0 %	50 %	100 %	0 %	50 %	100 %		
Group 1 (MeBT, BTA, IBS, CBZ, DFN, MTP, HCT)	*K*_F_	0.31	0.29	0.34	0.19	0.44	0.47	0.47	0.58
	*n*	−9.90	3.45	3.56	2.21	2.18	1.93	1.36	1.11
	*P*	0.81	0.22	0.11	0.01	0.03	0.04	0.01	0.04
	*R*^2^	0.04	0.61	0.79	0.97	0.94	0.92	0.99	0.93
	PAC demand (50 % reduction) (mg L^−1^)	28.4	13.8	12.1	15.8	6.6	5.5	3.6	2.1
	Factor (demand)	7.9	3.8	3.4	4.4	1.8	1.5	1.0	0.6
Group 2 (Group 1 + IBU, CDS, SMX)	*K*_F_	0.39	0.23	0.26	0.09	0.36	0.4	0.14	0.50
	*n*	−7.14	3.06	2.84	1.40	2.26	2.08	0.96	1.48
	*P*	0.82	0.39	0.09	0.01	0.01	0.01	0.01	0.01
	*R*^2^	0.03	0.37	0.83	0.99	0.99	0.99	0.99	0.99
	PAC demand (50 % reduction) (mg L^−1^)	37.1	20.6	17.3	24.5	10.2	8.4	6.4	4.4
	Factor (demand)	5.8	3.2	2.7	3.8	1.6	1.3	1.0	0.7
Sum OMPs total (Group 2 + ATA, IoHX, IoMP, IoPA, IoPR)	*K***_F_**	0.13	0.06	0.12	0.01	0.14	0.13	0.03	0.39
	*N*	3.52	1.26	1.77	0.75	1.44	1.26	0.69	1.53
	*P*	0.65	0.16	0.14	0.01	0.01	0.01	0.01	0.01
	*R*^2^	0.12	0.70	0.74	0.99	0.98	0.99	0.99	0.99
	PAC demand (50 % reduction) (mg L^−1^)	52.4^a^	30.0	26.2	40.6^a^	16.7	13.2	12.0	6.5
	Factor (demand)	4.4^a^	2.5	2.2	3.4^a^	1.4	1.1	1.0	0.6

a Extrapolated values noted for demands exceeding the isotherm range (maximum 40 mg L^−1^).

Group 3 included polar X-ray contrast agents, known to be challenging for conventional adsorption processes. In line with their high polarity and relatively large molecular structures, these compounds generally exhibited the lowest adsorption across all ACs. For instance, WET100 achieved a *K*_F_ of 0.13 (*R*^2^ = 0.99), whereas ORC100 was comparable at 0.12 (*R*^2^ = 0.74), but required twice as much PAC (26.2 mg L^−1^ versus 13.2 mg L^−1^) to reach 50 % removal. Among the reference ACs, WP 235 showed a notable advantage (*K*_F_ = 0.39, *R*^*2*^ = 0.99) and demanded only 6.5 mg L^−1^ for a 50 % reduction.

### 3.4 UV absorbance

**Fig. 4:**
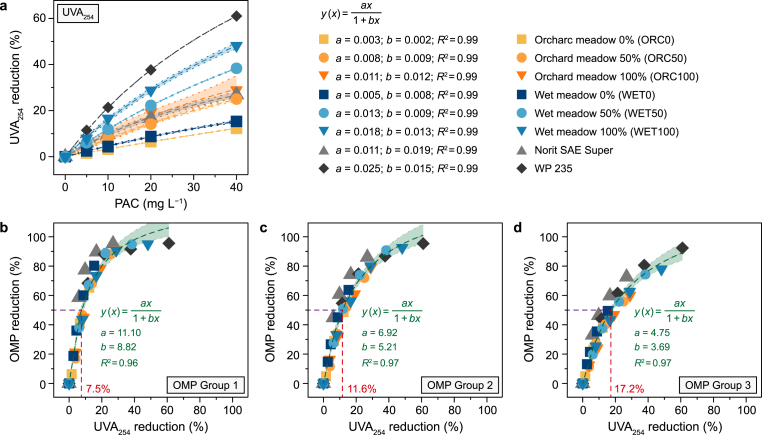
a, Normalized UV-absorbance at 254 nm (UVA_254_) reduction as a function of powdered activated carbon (PAC) dosage across AC types. Data are fitted with the model $y\left(x\right)=\frac{ax}{1+bx}$, where *y* is the UVA_254_ removal and *x* is the PAC dosage, yielding *R*^2^ = 0.99. Fitting parameters (*a*, *b*) are displayed for each biogenic AC type (orchard meadow 0–100 % [ORC0, ORC50, ORC100], wet meadow 0–100 % [WET0, WET50, WET100], as well as Norit SAE Super and WP 235). **b**–**d**, the correlation between normalized UVA_254_ reduction and organic micropollutant (OMP) removal for OMP Group 1 (**b**), Group 2 (**c**), and total OMPs (**d**), fitted with the model $y\left(x\right)=\frac{ax}{1+bx}$, where *y* is the OMP reduction, *x* is the UVA_254_ removal, and *R*^2^ the coefficient of determination. The horizontal dashed line (purple) represents 50 % OMP reduction, and the vertical dashed line (red) represents the corresponding UVA_254_ reduction. Shaded areas denote 95 % confidence intervals.

Normalized UVA_254_ values for ACs at different dosage levels showed a similar pattern to normalized OMP concentrations (Fig. 4a). For both biogenic ACs UVA_254_ reduction increased with increasing oxidation potential. Thereby, ORC ACs showed a notably lower reduction than ACs from WET biomass. In line with OMP removal, the highest UVA_254_ reduction was achieved by the conventional AC WP 235, while Norit SAE Super showed a substantially lower normalized UVA_254_ reduction compared to the OMP results and was in the range of ORC100 and ORC50.

Non-linear regression analysis between normalized UVA_254_ values and OMP reductions as response variable revealed a high correlation between both parameters, indicated by an *R*^2^ ≥ 0.96 and a narrow 95 % confidence interval. Thus, an OMP removal of 50 % corresponds to a UVA_254_ reduction of 7.5 % for OMP Group 1 (Fig. 4b), while the UVA_254_ value had to be reduced by roughly 11.6 % and 17.2 % for OMP Group 2 (Fig. 4c) and total OMPs (Fig. 4d), respectively. This is consistent with the results for the individual OMPs, except for X-ray contrast agents (Supplementary Fig. S6).

### 3.5 Greenhouse gas footprint of AC products

**Fig. 5:**
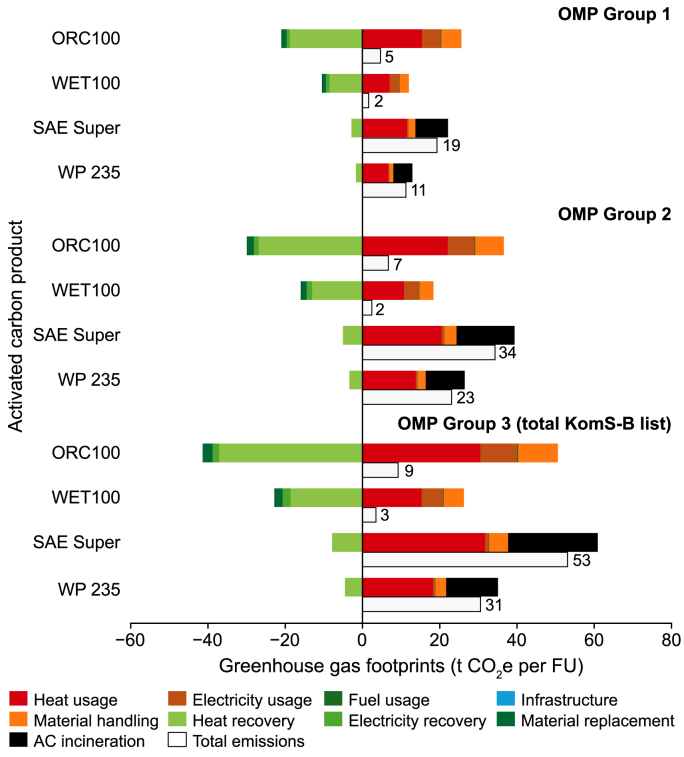
Greenhouse gas footprints associated with the production and use of activated carbon (AC) from orchard meadow (ORC100) and wet meadow (WET100) at 100 % activation, versus conventional ACs SAE Super and WP 235, across OMP Groups 1, 2, and 3 (total KomS-B list). Functional unit (FU): metric tonnes of AC needed for 50 % organic micropollutant (OMP) removal from 1000 m^3^ wastewater. Negative values indicate greenhouse gas savings, while positive values represent net emissions. Total emissions are depicted by the white bars.

The ‘gate to grave’ environmental performance of the LCM-based ACs to be applied for OMP Group 1 was estimated with a GHG footprint of 5 kg CO_2_ equivalent (CO_2_e) per FU, meaning per quantity (i.e., tonnes) of AC made from ORC that is needed to remove 50 % of OMPs from 1000 m^3^ of WW (Fig. 5). For the AC made from WET, a GHG footprint of 2 kg CO_2_e per FU was calculated. The footprint of the conventional standard ACs was estimated to be a total emission of 11 kg CO_2_e per FU, marketed under WP 235, and 19 kg CO_2_e per FU AC of the brand SAE Super. If we include the three pharmaceuticals ibuprofen, candesartan, and sulfamethoxazole, as well as the X-ray contrast agents to complete the KomS-B list (OMP Group 3), the GHG footprint of conventional standard ACs almost tripled, whereas the footprint of the ORC-based AC doubled, and that from WET barely increased. The GHG footprint of ACs for removing OMPs from the KomS-B list without X-ray contrast agent (OMP Group 2) revealed an intermediate total emission. Strongly influencing parameters on GHG footprint results were AC incineration (on the emission side) of conventional ACs, heat recovery (on the saving side; Supplementary Fig. S7) for ACs from LCM, as well as heat usage (also on the emission side), particularly for LCM press cake drying, and for activation of carbon products in general. Considering only the production of AC, the GHG footprint was estimated at 3.4 t CO_2_e per t AC for the conventional ACs and 0.81 or 0.85 t CO_2_e per t AC for ORC100 and WET100, respectively.

## 4 Discussion

### 4.1 Influence of physico-chemical characteristics of AC on OMP adsorption

Key parameters influencing AC efficiency in OMP adsorption include pore structure, chemical composition, ash content, mineral composition, and adsorption indices (e.g., iodine number) [37]. Combining these parameters enables a nuanced assessment of adsorption performance among the tested ACs and across different OMP groups.

Increasing oxidation levels from 0 % to 100 % in ACs from ORC and WET biomass enhanced SSA, meso- and micro-pore volume, and indicated iodine number, thereby improving adsorption capacity of the biogenic ACs [47]. Higher oxidation levels introduce oxygen-containing functional groups, which interact with polar OMPs through hydrogen bonding or π–π interactions, thereby enhancing OMP adsorption, particularly for most substances in this study, which contain aromatic rings in their molecular structures [[48], [49], [50]].

A comparison between ORC- and WET-derived ACs revealed intrinsic differences attributable to biomass composition. WET biomass, characterised by lower ash content and higher carbon content, yielded ACs with superior textural properties. This observation aligns with findings by Leng et al. [51], who demonstrated that a lower ash content in biomass precursors results in ACs with enhanced porosity due to reduced interference from inorganic matter during activation. Moreover, the higher methane yield observed from the anaerobic digestion of the WET press fluids further supports the notion that a lower inorganic burden in the feedstock leads to more efficient energy recovery and improved AC properties. ORC ACs exhibited higher concentrations of minerals, foremost Fe and Al, which can influence structural integrity and catalytic properties. Elevated mineral content may reduce porosity due to catalytic effects during pyrolysis, as noted by Tomczyk et al. [52] and Vijayaraghavan [53]. However, the high Fe and Al levels in ORC ACs did not impact the surface charge, as indicated by the zeta potential (Table 2; Supplementary Table S5). Measured at a pH of 8, one might expect Fe and Al to precipitate as insoluble hydroxides (Fe(OH)_3_ and Al(OH)_3_) rather than exist as free, positively charged ions, thereby contributing little to the surface charge. Moreover, if these metals were integrated within the carbon matrix, they would likely be less available at the surface to influence the zeta potential. In addition, the high ash content—rich in Ca, Mg, and K—could introduce competing minerals that might occupy adsorption sites or form compounds which would further reinforce the negative charge. Future studies should incorporate zeta potential measurements across a range of pH values (e.g., pH 4–10) to achieve a more comprehensive understanding of charge development, including the precise determination of the zero point of charge.

SAE Super and WP 235 had similar SSA, but WP 235 exhibited a slightly larger mesopore volume, potentially facilitating the adsorption of larger hydrophilic molecules in greater quantities by providing improved accessibility, extended diffusion pathways, and increased adsorption space [9]. This was particularly relevant for removing larger contaminants grouped under OMP Groups 2 and 3. Moreover, WP 235 had a higher iodine number, correlating with enhanced microporosity, thereby improving its capacity to adsorb small molecules [10,54], particularly relevant for OMP Group 1 compounds.

In evaluating the adsorption efficacy of WET100 in comparison to conventional ACs such as SAE Super and WP 235, WET100 demonstrated a commendable adsorption performance. Its surface chemistry is likely a key determinant of this efficacy. Compared to coal-derived materials, biogenic carbons typically exhibit a higher concentration of functional groups, such as hydroxyl, carboxyl, and phenolic groups [13]. These groups facilitate adsorption via hydrogen bonding, π–π interactions, and electrostatic forces [48,55], enhancing WET100's capacity for both hydrophilic and hydrophobic OMPs, particularly those containing sulfonic acid or hydroxyl groups, such as sulfamethoxazole, an antibiotic used to treat a variety of bacterial infections.

Its porous architecture with well-balanced mesoporosity and microporosity may facilitate synergistic interactions between pore structures and surface functionalities, thereby expanding its adsorption capacity [9]. Moreover, the WET biomass feedstock contributed to its performance, as biomass-derived carbons frequently retain organic functionalities that enhance surface reactivity [13]. This interplay between optimized porosity and inherent surface reactivity may have enabled WET100 to adsorb a broad spectrum of pollutants while exhibiting comparable or even superior efficiency to conventional ACs, particularly in the adsorption of larger or chemically complex molecules.

### 4.2 Specific OMP adsorption on AC and required quantity

The observed preference for Freundlich over Langmuir isotherms for both types of AC suggests heterogeneous adsorption processes, wherein AC surfaces exhibit diverse adsorption sites with varying affinities for the tested OMPs. The Freundlich model is often more suitable for describing adsorption onto biogenic AC, presumably due to its capacity to account for multiple adsorption mechanisms and dynamic interactions [56].

Variations in wastewater composition, competition for adsorption sites, potential pore blockage, and desorption effects [54,57] collectively complicate the quantitative comparison of ACs regarding their adsorption performance for specific OMPs in real wastewater matrices. Nevertheless, such assessments are essential for determining the required AC quantities to achieve a predefined removal threshold for OMPs. The primary challenge lies in the absence of a well-defined sum parameter to comprehensively evaluate overall OMP removal efficiency.

Our approach of grouping OMPs, as proposed by Kårelid et al. [58], not only facilitated a more concise representation of heterogeneous adsorption behaviour, but also allowed for including OMPs with ambiguous or limited adsorption tendencies, such as X-ray contrast agents. Designed for rapid renal excretion without interacting with cell membranes or proteins [59], contrast agents such as iomeprol and iohexol exhibited poor adsorption onto the tested ACs due to their high polarity and exceptional water solubility (Table 1; Supplementary Table S4 and Fig. S5). Their hydrophilic nature and relatively large molecular size impede diffusion into the micropores of ACs, where adsorption is most effective [10]. Moreover, electrostatic interactions remain minimal, as both the contrast agents and AC surfaces typically carry negative charges [48].

Nevertheless, such grouping may lead to overestimating or underestimating individual OMP removal and, consequently, the potentially associated environmental impacts. Incorporating freshwater ecotoxicological values specific to each OMP could mitigate these challenges by enabling the calculation of the overall ecotoxicity reduction achieved by different ACs [12]. However, such values are not available for all examined OMPs, introducing uncertainties into risk assessments, particularly as they fail to account for potential interactions between OMPs [60].

The adsorption performance of ACs for OMP removal was evaluated based on the Freundlich parameters (Table 3; Supplementary Fig. S5). The biogenic ORC100 exhibited the lowest adsorption capacity for OMPs, as reflected in its high *n*- and *p*-values, which indicate a lower affinity of adsorption sites and a more heterogeneous adsorption structure [56]. Furthermore, the Freundlich model demonstrated a weaker fit for ORC100, suggesting a less predictable adsorption behaviour. ORC100 proved highly inefficient, requiring the highest PAC dosage to achieve a 50 % reduction in OMP concentration. By contrast, WET100, derived from wet meadow biomass, exhibited a promising balance between adsorption capacity, efficiency, and material consumption compared to the conventional ACs Norit SAE Super and WP 235. Regarding Freundlich adsorption coefficient (*K*_F_), indicating adsorption capacity, WET100 displayed values that positioned it between SAE and WP 235, indicating competitive adsorption performance despite not being fossil-based. The adsorption intensity (*n*) of WET100 consistently exceeded that of SAE, although it remained below the performance level of WP 235.

A key factor for practical application is the resulting PAC demand. Across all OMP groups, WET100 required significantly less PAC to achieve a 50 % reduction in OMP concentration than ORC100 and was consistently positioned between SAE and WP 235. This suggests that AC derived from wet meadow biomass delivered adsorption performance comparable to SAE while maintaining moderate material consumption. Additionally, the model fit (*R*^2^) for WET100 remained consistently high (≥0.99) across all OMP groups, demonstrating a strong agreement with the Freundlich isotherm and confirming that the adsorption mechanisms were well described. In summary, WET100 exhibited remarkable efficiency for a biogenic, non-coal-based AC and represents a promising sustainable alternative for OMP removal in WWTP.

### 4.3 UVA_254_ for estimating total OMP reduction

A strong correlation between OMP removal and UVA_254_ reduction highlights their interdependence, endorsing UVA_254_ as a reliable surrogate for gauging overall OMP reduction, irrespective of the parent material's provenance (Supplementary Fig. S6). Although the UVA_254_ reduction was less marked than the corresponding OMP values, the relationship retains robustness. Nonetheless, as the UVA_254_ reduction associated with a 50 % OMP removal rises with the diversity of OMPs under consideration (Fig. 4), precise identification of the specific OMPs in the wastewater becomes essential. This observation concurs with previous studies that employed UVA_254_ measurements to predict OMP reduction and assess ACs' removal efficacy [15,[37], [38], [39]]. Moreover, the performance of ACs in terms of UVA_254_ reduction must be considered. For instance, conventional Norit SAE Super achieved considerably lower UVA_254_ removal than WET100 (Fig. 4a), despite similar overall OMP reduction (Fig. 2, Fig. 3). Comparison with other studies regarding UVA_254_ reduction is complicated because the initial OMP concentration in the wastewater was artificially elevated, thereby creating an imbalance with the natural organic background concentration. This imbalance may, in turn, result in an underestimation of OMP reduction at a given UVA_254_ threshold [61,62]. Despite the single effluent type tested, our results confirm that UVA_254_ is a valuable parameter for monitoring AC performance in wastewater treatment. It serves as a reliable proxy for predicting overall adsorption capacity. However, the inherent challenges in comparing adsorption performance and determining optimal dosages underscore the need for standardised protocols, particularly when considering environmental impacts such as greenhouse gas emissions and freshwater ecotoxicity.

### 4.4 Influence of biomass feedstock on the performance of biogenic ACs

The analysis of biomass composition and its impact on AC properties revealed significant differences between ORC and WET biomass, which can be attributed to their distinct site conditions and management practices. Orchard meadows, typically found on well-drained soils with moderate nutrient availability, are often managed extensively, involving periodic mowing or grazing [18]. This results in biomass with a relatively high ash content and a lower carbon fraction, negatively affecting pore development and adsorption capacity in the resulting ACs. The elevated mineral content, particularly by Ca, Fe, and Al, further influences the structural properties of the AC, reducing its porosity and surface area. In contrast, wet meadows are characterised by hydromorphic soils with higher organic matter accumulation due to water saturation and reduced decomposition rates [19]. These conditions bring about prolonged growth cycles and fibre-rich biomasses, resulting in a higher intrinsic carbon content and lower ash content in feedstock, eventually leading to advanced pore formation and enhanced adsorption properties. The AC derived from wet meadow biomass (WET100) exhibited a significantly larger specific surface area and greater micro- and mesopore volumes, directly enhancing adsorption efficiency. These findings highlight the critical role of feedstock selection and pretreatment in optimising AC performance for specific adsorption applications.

### 4.5 Greenhouse gas footprint of AC products

Based on the system boundaries suggested by Joseph et al. [12] and the promising performance of the WET100, using biogenic ACs for OMP removal was found to have a noticeably lower GHG footprint than conventional coal-based ACs. The pretreatment of biomass by IFBB proved pivotal for utilising LCM for AC production by reducing the feedstock's ash content and generating press fluid, thereby enhancing energy recovery. Notwithstanding the difficulty of some conventional ACs, such as the Norit SAE Super tested here, to achieve proper adsorption of X-ray contrast agents, the AC produced from wet meadow biomass proved to have a high potential to act as an effective adsorbent material for OMPs in WWTP at low environmental costs.

The European Commission issued draft revisions of the Urban Waste Water Treatment Directive (UWWTD 91/271) [63], introducing the concept of a “**fourth treatment**” (Supplementary Fig. S4). This new requirement mandates removing at least 80 % of indicator organic pollutants (KomS-B list) and will become compulsory for WWTPs serving ≥100000 population equivalents from 2035. Alt et al. [64] estimated that, if a fourth treatment stage were implemented across Germany, Austria, and Switzerland (commonly referred to as the D-A-CH region), approximately 41,000 metric tonnes of fossil-derived AC would be required annually. Since fossil coal-based AC production emits around 3.4 t CO_2_e per tonne, substituting it with biogenic AC derived from WET (with a performance factor of 1.1 relative to Norit SAE Super; Table 3) would save approximately 100000 t CO_2_ per annum. In light of these findings, reducing the global warming impact of advanced wastewater treatment in the long term can only be achieved through the sustainable replacement of fossil-based ACs with bio-based alternatives. Although our batch experiments—partly conducted with spiked OMPs over 24 h in a closed system—provided valuable insights into the adsorption performance and comparative GHG footprints of fossil-based versus biogenic ACs, we acknowledge that these conditions do not fully represent the continuous and dynamic environment of full-scale wastewater treatment, warranting further investigation under pilot-scale or continuous-flow conditions.

## 5 Conclusions

Our findings demonstrate that biogenic activated carbons produced from grassland biomass—specifically the wet meadow-based WET100 evaluated in this study—can effectively adsorb organic micropollutants in wastewater treatment. A dosage of approximately 13 mg L^−1^ of WET100 achieved 50 % OMP removal, equivalent to the performance of conventional activated carbons such as Norit SAE Super (12 mg L^−1^), but with a substantially lower greenhouse gas footprint. Importantly, our results highlight that standard AC parameters alone, such as surface area or pore volume, are insufficient for predicting real adsorption performance for OMP removal. Despite lower conventional values, WET100 excelled in OMP removal, highlighting the need for performance-based assessments when evaluating ACs for wastewater treatment applications. Future efforts should optimise biomass processing to reduce minerals and ash before carbonization, while refining pyrolysis parameters to enhance AC quality. This would help municipalities remediate water bodies from unwanted micro-pollutants by utilising untapped biogenic resources and promoting biodiversity-friendly grassland management regimes. Further research is needed to better understand adsorbate–adsorbent interactions, as OMPs remain a serious environmental and health concern.

## CRediT authorship contribution statement

**Korbinian Kaetzl:** Writing – review & editing, Writing – original draft, Investigation, Formal analysis, Data curation, Conceptualization, Methodology, Project administration, Supervision. **Marcel Riegel:** Writing – original draft, Funding acquisition, Investigation, Methodology. **Ben Joseph:** Writing – original draft, Investigation, Methodology. **Ronja Ossenbrink:** Writing – original draft, Investigation, Methodology. **Helmut Gerber:** Writing – review & editing, Funding acquisition, Conceptualization, Resources. **Willis Gwenzi:** Resources, Writing – review & editing. **Tobias Morck:** Writing – review & editing, Resources. **David Laner:** Writing – review & editing, Writing – original draft, Formal analysis. **Thomas Heinrich:** Writing – original draft, Investigation, Methodology. **Volker Kromrey:** Writing – review & editing, Conceptualization, Funding acquisition, Resources. **Kevin Friedrich:** Writing – review & editing, Conceptualization, Funding acquisition, Resources. **Michael Wachendorf:** Writing – review & editing, Conceptualization, Funding acquisition, Resources. **Kathrin Stenchly:** Writing – review & editing, Writing – original draft, Visualization, Formal analysis, Data curation, Validation.

## Data availability

Data will be made available on request.

## Declaration of competing interest

The authors declare that they have no known competing financial interests or personal relationships that could have appeared to influence the work reported in this paper.

## Appendix A Supplementary material

The Supporting Information contains details about biomass preparation, AC production, LCA scenario of AC production and utilization, wastewater and AC characteristics, and OMP adsorption isotherms.
