# Supplementary material for: Biogenic activated carbons from conservation grassland biomass for organic micropollutants removal in municipal wastewater
Source: Environ Sci Ecotechnol. 2025 Jun 6;26:100588. doi: 10.1016/j.ese.2025.100588 (PMC12210299; doi:10.1016/j.ese.2025.100588)
Supplement: Multimedia component 1 [file mmc1.pdf]

Supplementary material for

Biogenic Activated Carbons from Conservation Grassland Biomass for Organic Micropollutants Removal in Municipal Wastewater

Korbinian Kaetzl[1*], Marcel Riegel[2], Ben Joseph[3], Ronja Ossenbrink[1], Helmut Gerber[4], Willis Gwenzi[1,5,11], Tobias Morck[6], David Laner[7], Thomas Heinrich[8], Volker Kromrey[9], Kevin Friedrich[10], Michael Wachendorf[1] and Kathrin Stenchly[1]

[1]Section of Grassland Science and Renewable Plant Resources, University of Kassel, Steinstraße 19, 37213 Witzenhausen, Germany
[2]Section Concepts for Supply Structure, DVGW Technologiezentrum Wasser, Karlsruher Straße 84, 76139 Karlsruhe, Germany
[3]Thünen-Institute of Agricultural Technology, Bundesallee 47, 38116, Brunswick, Germany
[4]Pyreg GmbH, Trinkbornstraße 15-17, 56281 Dörth, Germany
[5]Department of Technology Assessment, Leibniz Institute for Agricultural Engineering and Bioeconomy, Max-Eyth-Allee 100, 14469 Potsdam, Germany
[6]Chair of Urban Water Engineering, University of Kassel, Kurt-Wolters-Straße 3, 34125, Kassel, Germany
[7]Research Center for Resource Management and Solid Waste Engineering, University of Kassel, Mönchebergstraße 7, 34125, Kassel, Germany
[8]Department of Postharvest Technologies, Leibniz Institute for Agricultural Engineering and Bioeconomy, Max-Eyth-Allee 100, 14469 Potsdam, Germany
[9]Bodensee-Stiftung, Fritz-Reichle-Ring 4, 78315 Radolfzell, Germany
[10]Björnsen Beratende Ingenieure GmbH, Branch Leonberg, Distelfeldstraße 15, 71229 Leonberg, Germany
[11]Biosystems and Environmental Engineering Research Group, 380 Adylin, Westgate, Harare, Zimbabwe
*Corresponding author: Korbinian Kaetzl (kaetzl@uni-kassel.de)

Table S1 Physico-chemical characteristics of landscape conservation material silage and generated press cake in the IFBB process.

		**Orchard Meadow (ORC)**		**Wet Meadow (WET)**	
**Parameter**	**Unit**	**Silage**	**Press cake**	**Silage**	**Press cake**
DM content	%FM	33.3	45.0	26.6	45.0
Ash content	%DM	19.4	9.6	5.4	3.2
Volatile Solids (VS)	%DM	80.6	90.4	95.6	96.7
C	%DM	40.7	45.9	48.0	49.4
O	%DM	33.7	37.9	39.8	40.8
H	%DM	4.8	5.4	5.5	5.6
N	%DM	1.4	1.3	1.1	1.0
S	%DM	0.09	0.08	0.18	0.03
Cl	%DM	0.10	0.05	0.68	0.02
ADF	g kg^{-1}DM	375	410	390	470
NDF	g kg^{-1}DM	582	655	565	671
ADL/Lignin	g kg^{-1}DM	128	99	95	108
Hemicellulose[a]	g kg^{-1}DM	207	245	175	201
Cellulose[a]	g kg^{-1}DM	247	321	295	362
HHV[a]	MJ kg^{-1}	16.5	18.2	19.0	19.6
LHV[a]	MJ kg^{-1}	15.6	17.2	18.0	18.5
DM flow in Press Cake	%	95		90	
DM flow in Press Fluid (PF)	%	5		10	
Methane yield (PF)	L_N CH_4 kg^{-1} VS	102		129	

[a]calculated; ADL = acid detergent lignin; ADF = acid detergent fibre; NDF = neutral detergent fibre, Hemicellulose = NDF − ADF, Cellulose = ADF − ADL.

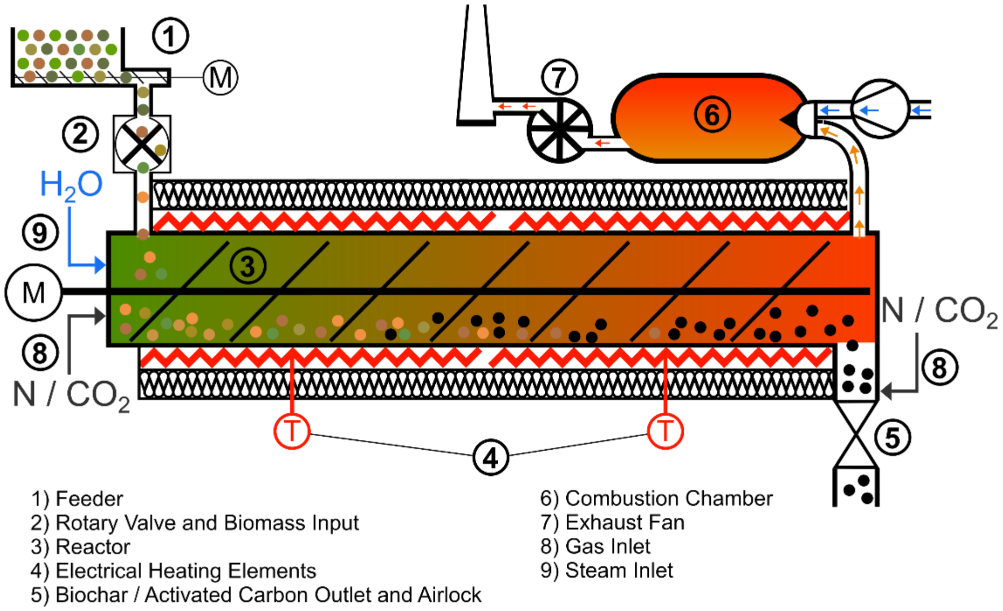

Fig. S1. Schematic drawing of the used laboratory pyrolysis and activation reactor.

The biogenic activated carbons (ACs) investigated in this study were produced in a continuously operated auger laboratory reactor (Pyreka, Pyreg GmbH, Dörth, Germany). The reactor was electrically heated, with the temperature set to 900 °C. Biomass pellets were fed into the reactor via a screw conveyor, ensuring a constant and controlled input stream. The material was transported through the reactor with a residence time of 25 minutes. At the reactor's outlet, the resulting AC was collected in an airtight container, while the syngas generated during the process was completely incinerated in the combustion chamber (Fig. S1). Activation was conducted by direct injection of H_2O steam into the pyrolysis process. To ensure an optimal comparability of ACs produced from different biomass feedstocks, the amount of added oxygen was stoichiometrically normalized to the carbon content of the biomass. From each biomass, three ACs were produced by adding the stoichiometric amount of oxygen to oxidize 0 %, 50 % and 100 % of carbon content of each biomass. AC from the run-in and run-out of the reactor were disposed to ensure steady-state conditions for AC production in the reactor. Conversion rate of each AC was determined by measuring input and output (Table S2).

Table S2 Pyrolysis and activation conditions for producing biogenic activated carbon from biomass collected from orchard meadows (ORC) and wet meadows (WET), including biomass carbon content (C), dry matter (DM) input, carbon feed rate (g h^{-1}, mol h^{-1}), H_2O and O_2 addition for defined activation levels, and the corresponding conversion rate.

**Sample**	**Temp.** [°C]	**Residence time** [min]	**C** [%DM]	**DM** [g h^{-1}]	**C** [g h^{-1}]	**C** [mol h^{-1}]	**H_2O** [mL h^{-1}]	**O** [mol h^{-1}]	**Activation** [%]	**Conversion rate** [%DM]
ORC0	900	25	45	2,619	1,179	98	0	0	0	22.6
ORC50	900	25	45	2,600	1,170	97	889	49	50	19.1
ORC100	900	25	45	2,755	1,240	103	1,735	93	100	17.2
WET0	900	25	48	2,995	1,438	120	0	0	0	16.9
WET50	900	25	48	2,946	1,414	118	1,075	51	50	14.3
WET100	900	25	48	2,312	1,110	92	1,588	95	100	11.7

Table S3. Limit of quantification, initial concentrations of OMPs, concentrations after spiking, and characteristics of the wastewater used in this study, obtained from the effluent of the wastewater treatment plant in Kressbronn-Langenargen, Germany.

**Substance**	**Initial concentration** [µg/L]	**Concentration after spiking** [µg/L]	**Limit of quantification [µg/L]**
Benzotriazole (BTA)	2.65	4.3	0.1
Carbamazepine (CBZ)	0.17	2.15	0.1
Diclofenac (DFN)	0.87	1.95	0.1
Ibuprofen (IBU)	< 0.1	2.58	0.1
Hydrochlorothiazide (HCT)	0.5	2.0	0.1
Amidotrizoic acid (ATA)	0.18	2.45	0.1
Sulfamethoxazole (SMX)	0.056	1.65	0.1
Metoprolol (MTP)	0.38	2.2	0.1
Irbesartan (IBS)	0.17	1.25	0.1
Iopromide (IoPR)	< 0.1	1.5	0.1
Iomeprol (IoMP)	0.64	0.82	0.1
Candesartan (CDS)	1.05	2.5	0.1
Iohexol (IoHX)	2.1	2.05	0.1
Iopamidol (IoPA)	< 0.1	1.85	0.1
Σ 4(5)-Methylbenzotriazole (MeBT)	0.54	1.05	0.1
**Parameters**	[mg/L]		
Total Solids (TS)	43.5		
Total Suspended Solids (TSS)	5.8		
Chemical Oxygen Demand (COD)	19.1		
Dissolved Organic Carbon (DOC)	27.0		0.2

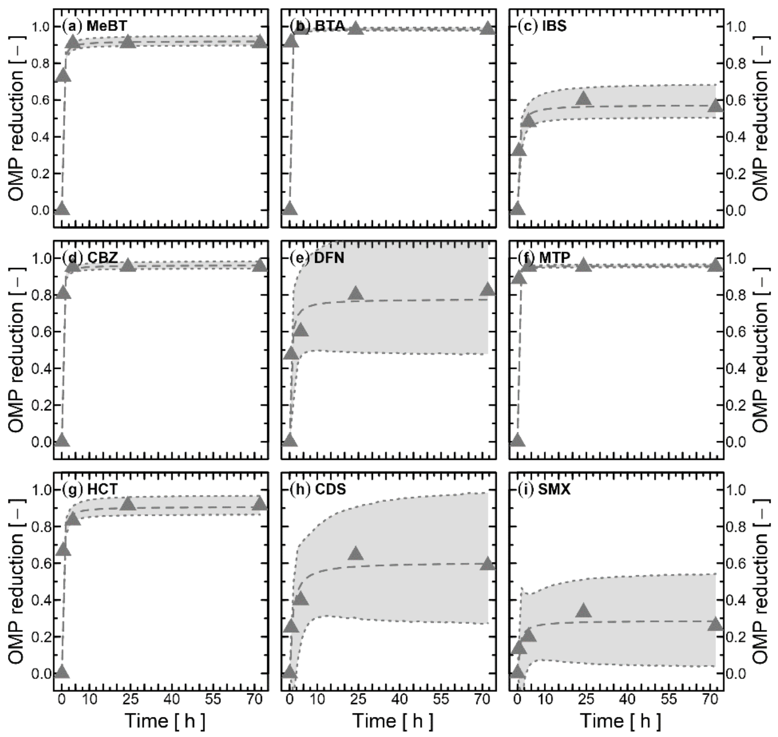

Fig. S2. Reduction of Σ 4(5)-Methylbenzotriazole (MeBT), Benzotriazole (BTA), Irbesartan (IBS), Carbamazepine (CBZ), Diclofenac (DFN), Metropolol (MTP), Hydrochlorothiazide (HCT), Candesartan (CDS) and Sulfamethoxazole (SMX) by the PAC Norit SAE Super at different contact times. Dashed line represents the calculated regression line and the shaded area the calculated 95 % confidence interval.

Kinetic studies were conducted on nine OMPs using the commercial reference PAC Norit SAE Super to validate literature findings on equilibrium concentration (Ce). A PAC dosage of 20 mg L^{-1} was applied with contact times of 0, 0.5, 4, 24, and 72 hours (Fig. S2). Results showed that 24 hours was sufficient to reach equilibrium and was used for the main adsorption experiments.

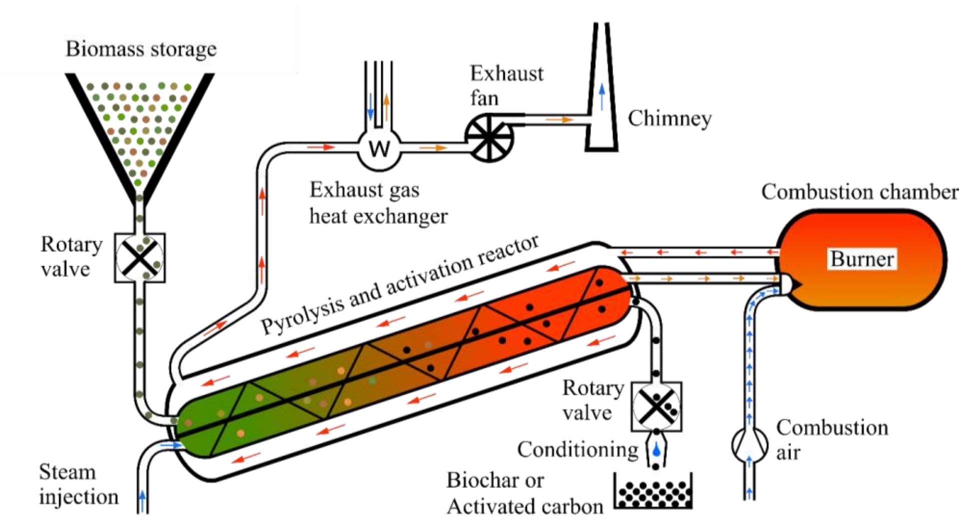

Fig. S3. Schematic flow diagram of the PYREG A500 pyrolysis and activation unit illustrating important process steps along with inputs and outputs.

To assess the global warming potential (GWP) of biogenic activated carbon via life cycle assessment (LCA), a full-scale pyrolysis and activation reactor was considered as a potential implementation scenario.

A pilot-scale reactor (A500, Pyreg GmbH, Dörth, Germany) was installed at the Baden-Baden wastewater treatment plant (Germany) for producing activated carbon (AC) and biochar from residual biomass. In this reactor, dried biomass undergoes high-temperature thermal treatment in an oxygen-free environment, releasing volatile compounds as pyrolysis gas, which is incinerated at 1,200 °C in a FLOX burner. This prevents polycyclic aromatic hydrocarbon (PAH) formation by decomposing harmful substances. The resulting exhaust gases heat the reactor.

Operating at up to 900 °C, the reactor enables partial carbon oxidation in the presence of H_2O steam. Excess heat is recoverable via a heat exchanger for processes such as steam generation or feedstock drying. Rotary wheel sluices at the inlet and outlet prevent oxygen intrusion, while steam injection facilitates AC production through physical activation. Pyrolysis gas combustion ensures compliance with the German Emission Control Act.

Designed for continuous operation and diverse feedstocks, the reactor processes 750–1,000 t dry matter per year, depending on carbon content, particle size, and process parameters (e.g., pyrolysis temperature, retention time). By utilizing pyrolysis gas for heating and steam generation, the process is energy self-sufficient, producing up to 150 kW surplus thermal power (~1 GWh/a). The A500 reactor allows adjustable pyrolysis temperatures (500–900 °C) and defined solid retention times. Steam addition enables controlled biomass oxidation, enhancing AC surface area and adsorption capacity.

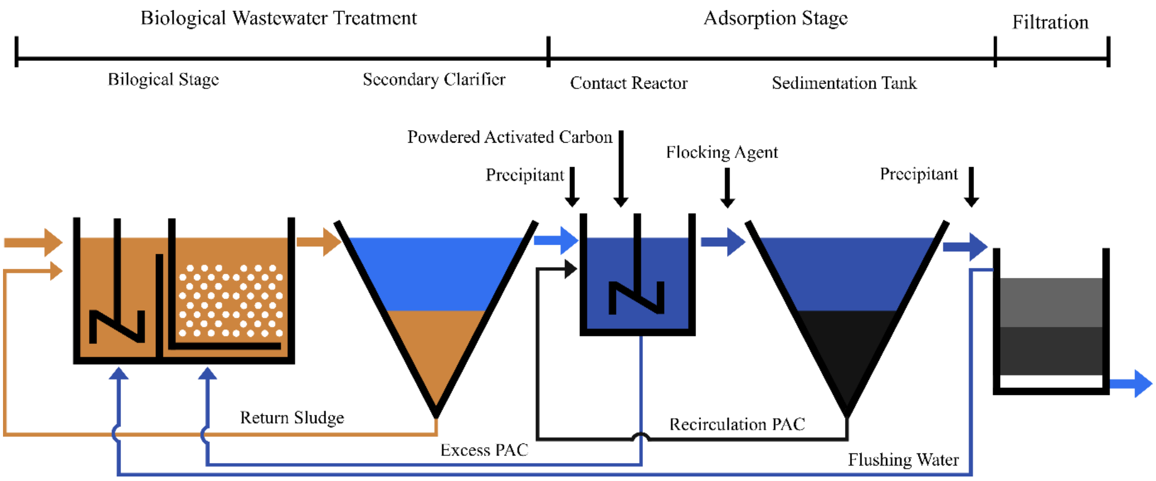

Fig. S4. Schematic diagram of a wastewater treatment plant for OMP removal by addition of powdered activated carbon (PAC) as a fourth treatment stage in a downstream contact reactor according to Eidgenössische Anstalt für Wasserversorgung, Abwasserreinigung und Gewässerschutz (2010).

Activated carbon is widely used for the removal of organic micropollutants (OMPs) from municipal wastewater (WW). In this study, the application of powdered activated carbon (PAC) in a contact reactor following biological wastewater treatment was assumed.

For the adsorption experiments, wastewater was sourced from the municipal wastewater treatment plant (WWTP) Kressbronn-Langenargen (Baden-Württemberg, Germany), which serves a population equivalent of 25,600. This WWTP is a four-stage treatment facility that already employs PAC for OMP removal. The treatment process consists of mechanical separation via a screening system, followed by a grit chamber and a primary clarifier (first stage). In the second stage, carbon and nitrogen compounds are biologically degraded in the aeration tank, while phosphorus is chemically precipitated in the third stage.

In the fourth stage, PAC is introduced into a contact reactor, where it is mixed with the effluent from the secondary clarifier in the presence of a flocculation agent. After a contact time of approximately 30 minutes, the PAC and treated wastewater are separated in a sedimentation tank, and the treated effluent is discharged into Lake Constance. To enhance OMP removal, settled PAC is recycled into the biological treatment stage, ensuring maximum adsorption efficiency (Boehler et al. 2012). The spent PAC is ultimately removed together with the excess sludge in the secondary clarifier and incinerated along with the digested sludge.

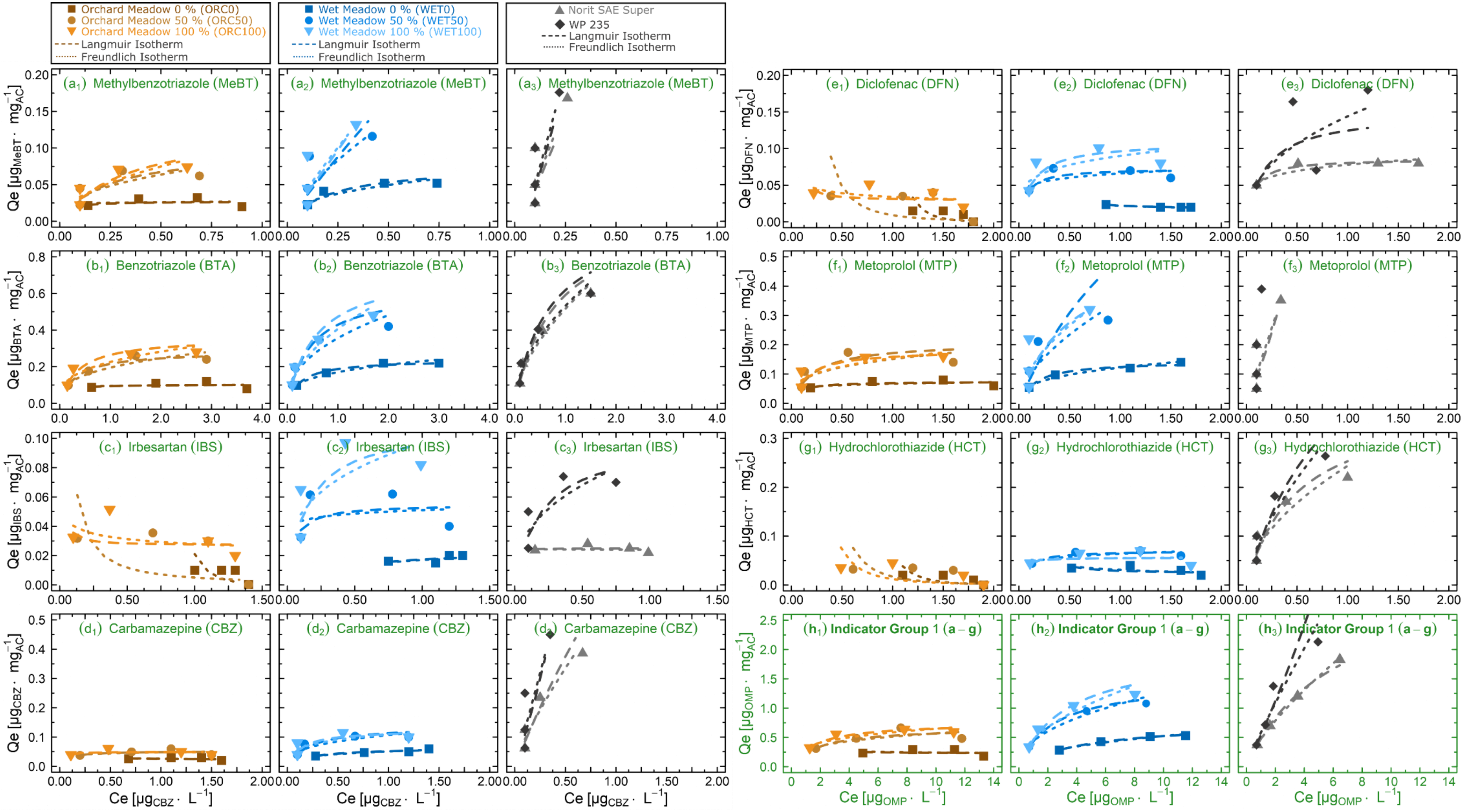

Fig. S5a. Freundlich (···) and Langmuir (---) isotherms for OMP group 1 (a-g) and sum of OMP group 1 (h) for AC from orchard meadow (ORC; index 1), wet meadow (WET; index 2) and conventional ACs Norit SAE Super and WP 235 (index 3).

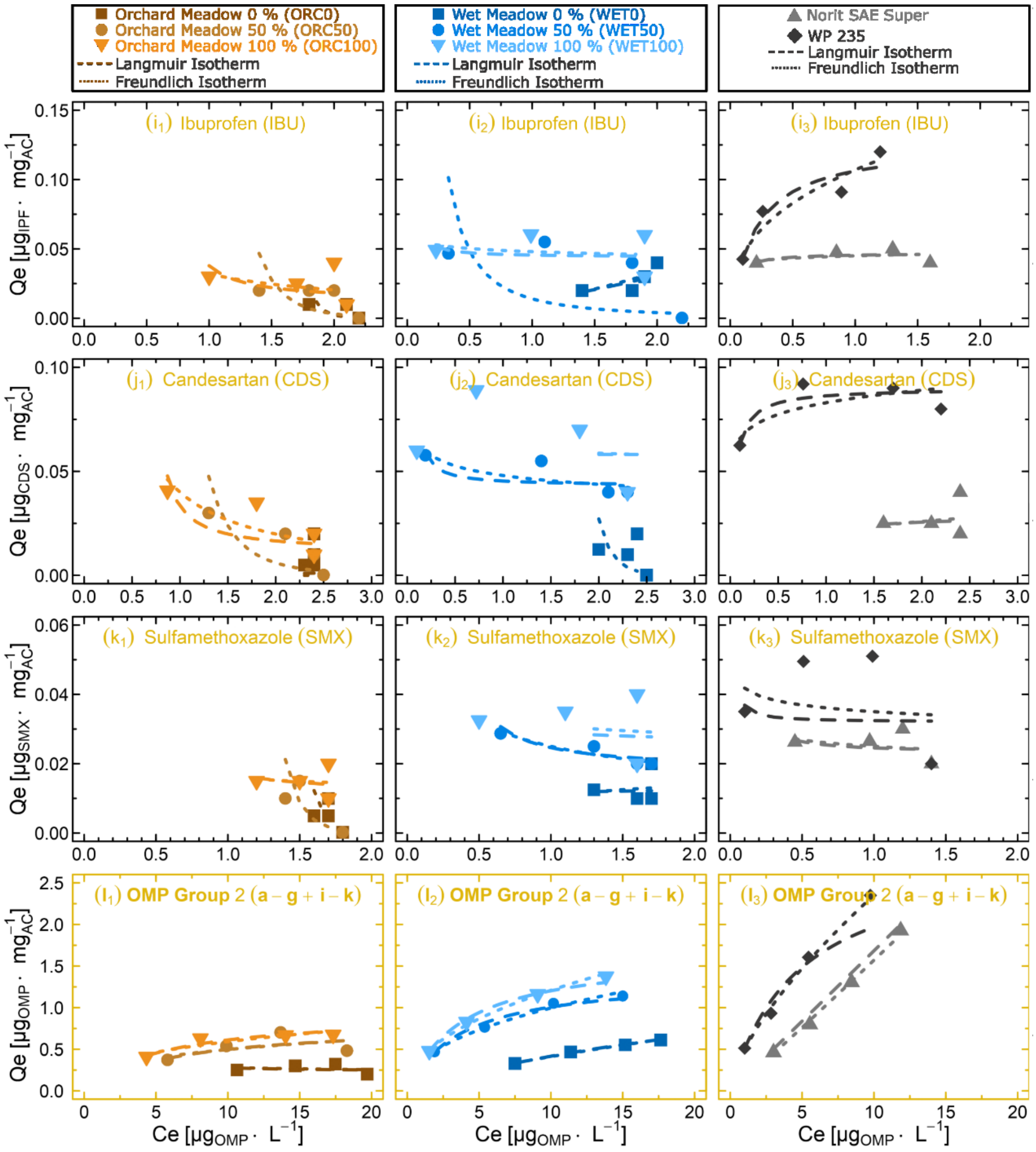

Fig. S5b. Freundlich (···) and Langmuir (---) isotherms for OMP group 2 (i-k) and sum of OMP group 1 and group 2 (l) for AC from orchard meadow (ORC; index 1), wet meadow (WET; index 2) and conventional ACs Norit SAE Super and WP 235 (index 3).

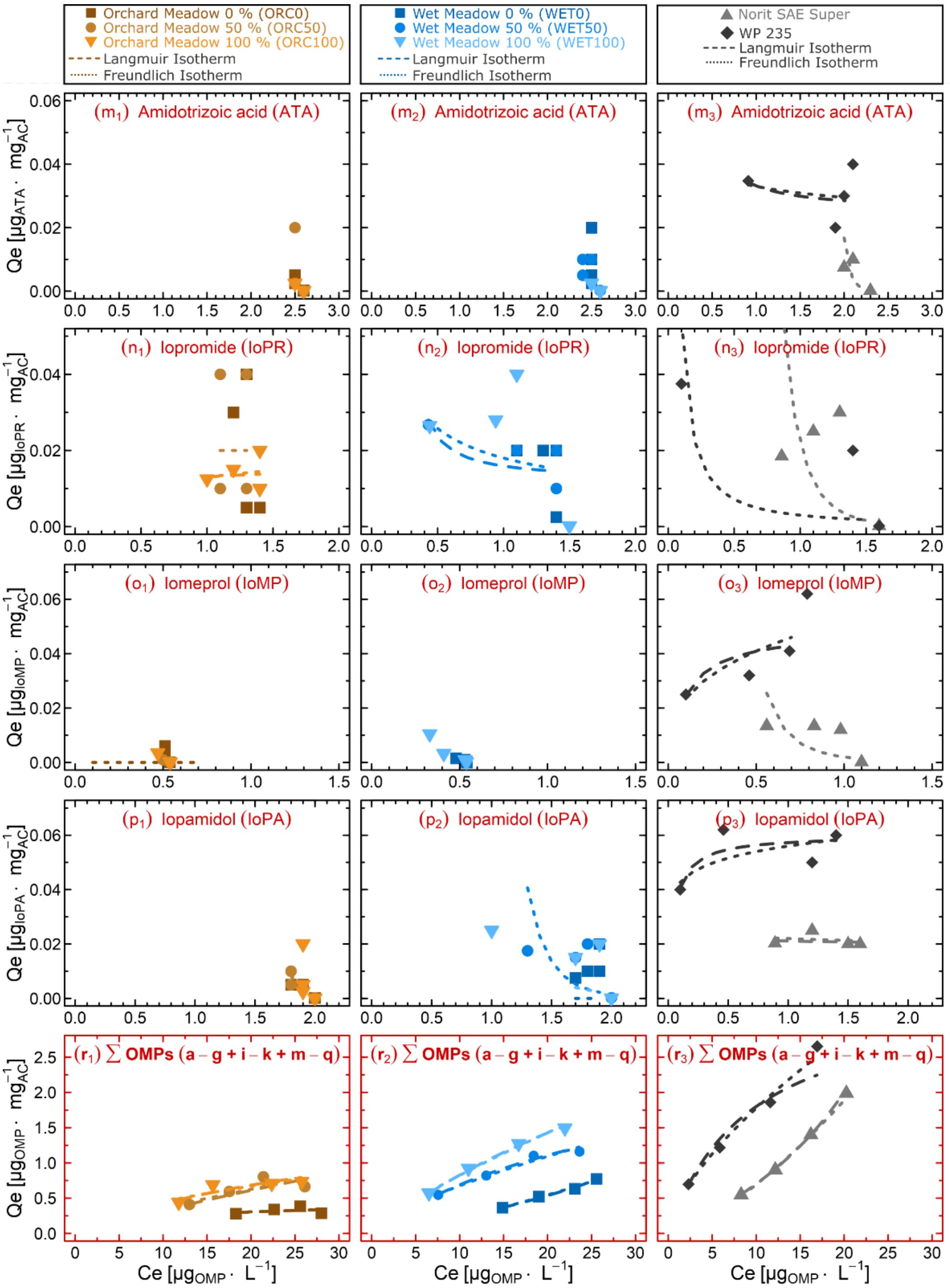

Fig. S5c. Freundlich (···) and Langmuir (---) isotherms for OMP group 3 (m-p) and sum of KomS-B list (r) including OMP group 1 and group 2 for AC from orchard meadow (ORC; index 1), wet meadow (WET; index 2) and conventional ACs Norit SAE Super and WP 235 (index 3).

Freundlich and Langmuir coefficients were determined for all OMPs, PACs, and OMP groups (Table S4). However, neither model adequately described the adsorption behavior of all OMPs on each AC, and in some cases, coefficients could not be determined—particularly for most X-ray contrast agents and biogenic AC with low adsorption capacity.

Table S4 Langmuir and Freundlich coefficients for Benzotriazole (BTA), Diclofenac (DFN) Carbamazepine (CBZ), Irbesartan (IBS), Metoprolol (MTP), Methylbenzotriazole (MeBT), Hydrochlorothiazide (HCT), OMP group 1, Ibuprofene (IBU), Candesartan (CDS), Sulfamethoxazole (SMX), OMP group 2, Amidotrizoic acid (ATA), Iopromide (IoPR), Iomeprol (IoMP), Iopamidol (IoPA), Iohexol (IoHX), Sum of OMP total.

			Activated Carbon							
**OMP**	**Equation**	**Coefficient**	**SAE**	**WP235**	**ORC0**	**ORC50**	**ORC100**	**WET0**	**WET50**	**WET100**
**BTA**	**Langmuir**	K_L	1.24	1.46	10.35	3.09	2.78	3.61	2.12	1.73
		q_{max}	1.07	1.04	0.10	0.29	0.36	0.24	0.63	0.75
		p	0.02	0.10	0.71	0.01	0.04	0.00	0.05	0.04
		R^2	0.95	0.82	0.09	0.99	0.92	0.99	0.91	0.92
	**Freundlich**	K_F	0.52	0.53	0.10	0.20	0.23	0.17	0.36	0.41
		**n**	1.71	1.83	21.74	2.96	3.12	3.24	2.30	1.98
		p	0.03	0.65	0.81	0.05	0.10	0.02	0.07	0.05
		R^2	0.94	0.87	0.04	0.90	0.82	0.97	0.86	0.91
**DFN**	**Langmuir**	K_L	14.08	5.10	ND	ND	-13.65	ND	15.00	7.57
		q_{max}	0.09	0.15	0.00	0.00	0.03	0.00	0.07	0.11
		p	0.01	0.18	0.43	0.55	0.66	0.43	0.12	0.13
		R^2	0.98	0.67	0.32	0.20	0.12	0.32	0.78	0.75
	**Freundlich**	K_F	0.08	0.14	0.16	0.01	0.03	0.16	0.07	0.09
		**n**	5.95	2.30	-0.11	-0.41	-4.93	-0.11	7.58	4.72
		p	0.08	0.27	0.36	0.45	0.54	0.36	0.34	0.29
		R^2	0.85	0.54	0.42	0.31	0.21	0.42	0.44	0.50
**CBZ**	**Langmuir**	K_L	0.31	-624.00	-9.90	14.03	43.67	4.55	4.57	7.49
		q_{max}	2.76	-1.61	0.02	0.05	0.05	0.06	0.14	0.13
		p	0.17	0.42	0.79	0.39	0.57	0.04	0.06	0.28
		R^2	0.69	0.34	0.05	0.37	0.19	0.93	0.89	0.52
	**Freundlich**	K_F	0.58	1.32	0.03	0.05	0.05	0.05	0.11	0.11
		**n**	1.25	0.98	-7.81	8.70	34.48	3.45	3.02	3.65
		p	0.08	0.25	0.75	0.52	0.83	0.03	0.13	0.27
		R^2	0.84	0.56	0.07	0.23	0.19	0.93	0.76	0.53
**IBS**	**Langmuir**	K_L	303.03	5.04	ND	-8.79	-41.34	2.27	-7.94	6.57
		q_{max}	0.03	0.10	0.00	0.00	0.03	0.03	0.00	0.11
		p	0.91	0.26	0.38	0.61	0.69	0.47	0.44	0.28
		R^2	0.01	0.55	0.38	0.16	0.10	0.28	0.31	0.52
	**Freundlich**	K_F	0.02	0.09	0.02	0.01	0.03	0.02	0.01	0.10
		**n**	-142.86	2.59	-0.19	-1.19	-6.29	2.80	-1.01	3.29
		p	0.94	0.23	0.35	0.48	0.51	0.40	0.41	0.28
		R^2	0.00	0.59	0.42	0.27	0.24	0.35	0.35	0.51
**MeBT**	**Langmuir**	K_L	-1.74	-2.78	35.04	4.55	3.00	4.46	0.24	-0.03
		q_{max}	-0.20	-0.11	0.03	0.10	0.13	0.08	1.57	-14.29
		p	0.43	0.42	0.69	0.27	0.23	0.04	0.40	0.45
		R^2	0.33	0.33	0.10	0.54	0.59	0.92	0.36	0.31
	**Freundlich**	K_F	0.93	1.97	0.03	0.08	0.11	0.07	0.23	0.34
		**n**	0.79	0.63	20.41	2.51	1.99	2.55	1.39	1.14
		p	0.27	0.26	0.83	0.26	0.19	0.10	0.31	0.31
		R^2	0.53	0.55	0.03	0.55	0.66	0.82	0.47	0.48
**MTP**	**Langmuir**	K_L	-0.67	-5.72	13.61	4.54	6.75	6.15	0.64	2.15
		q_{max}	-1.20	-0.06	0.07	0.21	0.18	0.14	1.27	0.53
		p	0.42	0.41	0.25	0.15	0.25	0.00	0.21	0.45
		R^2	0.33	0.35	0.56	0.73	0.56	0.99	0.62	0.30
	**Freundlich**	K_F	1.07	227.51	0.07	0.15	0.15	0.12	0.35	0.39
		**n**	0.97	0.30	9.26	3.46	3.44	3.10	1.72	1.82
		p	0.26	0.23	0.42	0.25	0.19	0.02	0.18	0.31
		R^2	0.55	0.59	0.34	0.57	0.65	0.97	0.67	0.47
**HCT**	**Langmuir**	K_L	1.99	1.11	-0.61	-1.99	-1.45	-4.88	13.46	42.22
		q_{max}	0.38	0.67	0.00	0.00	0.00	0.02	0.07	0.06
		p	0.14	0.18	0.32	0.46	0.50	0.49	0.06	0.67
		R^2	0.74	0.67	0.47	0.29	0.25	0.26	0.88	0.11
	**Freundlich**	K_F	0.24	0.35	0.05	0.02	0.02	0.03	0.06	0.06
		**n**	1.86	1.50	-0.25	-0.47	-0.54	-3.09	6.71	23.81
		p	0.10	0.10	0.17	0.35	0.35	0.39	0.19	0.81
		R^2	0.82	0.81	0.69	0.43	0.43	0.37	0.66	0.04

**OMP group 1**	**Langmuir**	K_L	0.17	-0.03	-1.75	0.52	0.61	0.21	0.42	0.30
		q_{max}	3.36	-15.87	0.22	0.68	0.79	0.77	1.46	1.98
		p	0.00	0.01	0.85	0.09	0.03	0.00	0.00	0.01
		R^2	0.99	0.97	0.02	0.84	0.95	1.00	0.99	0.98
	**Freundlich**	K_F	0.47	0.58	0.31	0.29	0.34	0.19	0.44	0.47

		**n**	1.36	1.11	-9.90	3.45	3.56	2.21	2.18	1.93
		p	0.00	0.04	0.81	0.22	0.11	0.01	0.03	0.04
		**R^2**	1.00	0.93	0.04	0.61	0.79	0.97	0.94	0.92
**CDS**	**Langmuir**	**K_L**	2.89	23.29	-0.22	0.22	-1.49	-1.49	-19.17	-161.13
		**q_{max}**	0.03	0.09	0.00	0.00	0.01	0.01	0.04	0.06
		p	0.89	0.07	0.44	0.44	0.39	0.39	0.32	0.92
		**R^2**	0.01	0.87	0.31	0.00	0.38	0.38	0.47	0.01
	**Freundlich**	**K_F**	0.02	0.08	0.00	0.00	0.04	0.04	0.05	0.06
		**n**	3.30	9.90	0.06	0.06	-0.99	-0.99	-7.52	-18.18
		p	0.80	0.20	0.48	0.48	0.24	0.24	0.22	0.77
		**R^2**	0.04	0.64	0.27	0.00	0.58	0.58	0.61	0.05
**IBU**	**Langmuir**	**K_L**	29.47	5.10	ND	-0.40	ND	-0.16	-4.04	-29.48
		**q_{max}**	0.05	0.13	0.00	0.00	0.00	-0.07	0.00	0.04
		p	0.47	0.01	0.22	0.48	0.22	0.27	0.47	0.82
		**R^2**	0.28	0.98	0.60	0.26	0.60	0.54	0.28	0.03
	**Freundlich**	**K_F**	0.05	0.11	4.49	0.07	4.49	0.01	0.01	0.05
		**n**	18.18	2.73	-0.10	-0.22	-0.10	0.63	-0.78	-14.93
		p	0.58	0.04	0.21	0.49	0.21	0.25	0.50	0.80
		**R^2**	0.18	0.91	0.62	0.26	0.62	0.56	0.25	0.04
**SMX**	**Langmuir**	**K_L**	-15.79	-75.67	ND	-0.50	-2.70	8.97	-3.72	-9.32
		**q_{max}**	0.02	0.03	0.00	0.00	0.01	0.01	0.02	0.03
		p	0.73	0.87	0.35	0.31	0.82	0.97	0.12	0.76
		**R^2**	0.07	0.02	0.43	0.48	0.03	0.00	0.77	0.06
	**Freundlich**	**K_F**	0.03	0.04	0.26	0.10	0.02	0.01	0.03	0.03
		**n**	-9.80	-12.99	-0.13	-0.18	-5.81	3.15	-2.66	-8.48
		p	0.70	0.79	0.47	0.29	0.90	0.88	0.07	0.79
		**R^2**	0.09	0.04	0.28	0.50	0.01	0.02	0.87	0.05
**OMP group 2**	**Langmuir**	**K_L**	0.00	0.21	-0.58	0.16	0.19	0.03	0.27	0.26
		**q_{max}**	-167.00	2.92	0.02	0.81	0.94	1.68	1.39	1.67
		p	0.00	0.01	0.83	0.24	0.04	0.00	0.01	0.00
		**R^2**	1.00	0.98	0.03	0.57	0.93	1.00	0.99	1.00
	**Freundlich**	**K_F**	0.14	0.50	0.39	0.23	0.26	0.08	0.36	0.40
		**n**	0.96	1.48	-7.14	3.06	2.84	1.40	2.26	2.08
		p	0.00	0.00	0.82	0.39	0.09	0.00	0.01	0.00
		**R^2**	1.00	0.99	0.03	0.37	0.83	0.99	0.99	0.99
**ATA**	**Langmuir**	**K_L**	ND	-4.05	ND	ND	ND	ND	ND	-4.05
		**q_{max}**	0.00	0.03	0.00	0.00	0.00	0.00	0.00	0.03
		p	0.14	0.73	0.10	0.10	0.33	0.33	0.15	0.73
		**R^2**	0.74	0.07	0.81	0.81	0.45	0.00	0.73	0.07
	**Freundlich**	**K_F**	>>100	0.03	>>100	>>100	>>100	0.01	>>100	0.03
		**n**	-0.03	-6.25	-0.01	-0.01	-0.01	-0.01	-0.03	-6.25
		p	0.06	0.79	0.02	0.05	0.06	0.06	0.19	0.79
		**R^2**	0.88	0.05	0.96	0.90	0.89	0.00	0.65	0.05
**IoPR**	**Langmuir**	**K_L**	ND	ND	-0.72	ND	4.43	-1.16	-4.07	ND
		**q_{max}**	0.00	0.00	0.00	0.02	0.02	0.00	0.01	0.00
		p	0.30	0.41	0.31	1.00	0.91	0.55	0.43	0.48
		**R^2**	0.49	0.35	0.47	0.00	0.01	0.21	0.33	0.27
	**Freundlich**	**K_F**	0.02	0.00	0.27	0.02	0.01	0.04	0.02	0.01
		**n**	-0.16	-0.80	-0.09	ND	3135.00	-0.24	-1.95	-0.34
		p	0.29	0.27	0.35	1.00	0.83	0.54	0.31	0.40
		**R^2**	0.50	0.53	0.42	0.00	0.03	0.21	0.48	0.36
**IoMP**	**Langmuir**	**K_L**	ND	10.05	ND	ND	ND	ND	ND	ND
		**q_{max}**	0.00	0.05	0.00	0.00	0.00	0.00	0.00	0.00
		p	0.45	0.16	0.10	0.35	0.10	0.10	0.10	0.34
		**R^2**	0.30	0.71	0.82	0.42	0.82	0.82	0.82	0.44
	**Freundlich**	**K_F**	0.00	0.05	0.00	0.00	0.00	0.00	0.00	0.00
		**n**	-0.23	2.90	-0.01	-0.03	-0.01	-0.01	-0.01	-0.15
		p	0.38	0.16	0.01	0.20	0.10	0.10	0.10	0.07
		**R^2**	0.39	0.71	0.98	0.81	0.98	0.98	0.98	0.86
**IoAP**	**Langmuir**	**K_L**	-19.01	20.96	ND	ND	ND	-0.53	ND	ND
		**q_{max}**	0.02	0.06	0.00	0.00	0.00	0.00	0.00	0.00
		p	0.88	0.14	0.20	0.10	0.00	0.20	0.41	0.56
		**R^2**	0.02	0.75	0.64	0.81	1.00	0.65	0.35	0.19
	**Freundlich**	**K_F**	0.02	0.06	>>100	>>100	>>100	0.00	0.30	0.04
		**n**	-12.99	8.48	-0.03	-0.03	-0.01	0.17	-0.13	-0.23
		p	0.81	0.29	0.11	0.02	0.08	0.27	0.37	0.48
		**R^2**	0.04	0.50	0.79	0.97	0.85	0.54	0.39	0.27

**IoHX**	**Langmuir**	**K_L**	0.78	8.37	-0.43	-0.69	-3.82	-0.28	ND	0.53
		**q_{max}**	0.06	0.14	-0.01	0.00	0.02	-0.04	0.00	0.15
		p	0.17	0.28	0.07	0.02	0.92	0.60	0.59	0.27
		**R^2**	0.69	0.52	0.86	0.96	0.01	0.16	0.17	0.53
	**Freundlich**	**K_F**	0.03	0.12	0.00	0.00	0.03	0.02	0.04	0.05
		**n**	1.86	3.75	0.27	0.10	-2.47	0.53	-0.26	1.21
		p	0.18	0.21	0.05	0.08	0.83	0.69	0.39	0.26
		**R^2**	0.67	0.62	0.91	0.85	0.03	0.10	0.38	0.56
**Sum OMP total**	**Langmuir**	**K_L**	-0.02	0.11	0.10	0.01	0.03	-0.01	0.03	0.02
		**q_{max}**	-2.35	3.50	0.46	4.96	1.83	-1.53	2.84	5.18
		p	0.00	0.01	0.60	0.10	0.10	0.00	0.00	0.00
		**R^2**	1.00	0.98	0.16	0.82	0.80	0.99	0.99	1.00
	**Freundlich**	**K_F**	0.03	0.39	0.13	0.06	0.12	0.01	0.14	0.13
		**n**	0.69	1.53	3.52	1.26	1.77	0.75	1.44	1.26
		p	0.00	0.00	0.65	0.16	0.14	0.01	0.01	0.00
		**R^2**	1.00	0.99	0.12	0.70	0.74	0.99	0.98	0.99

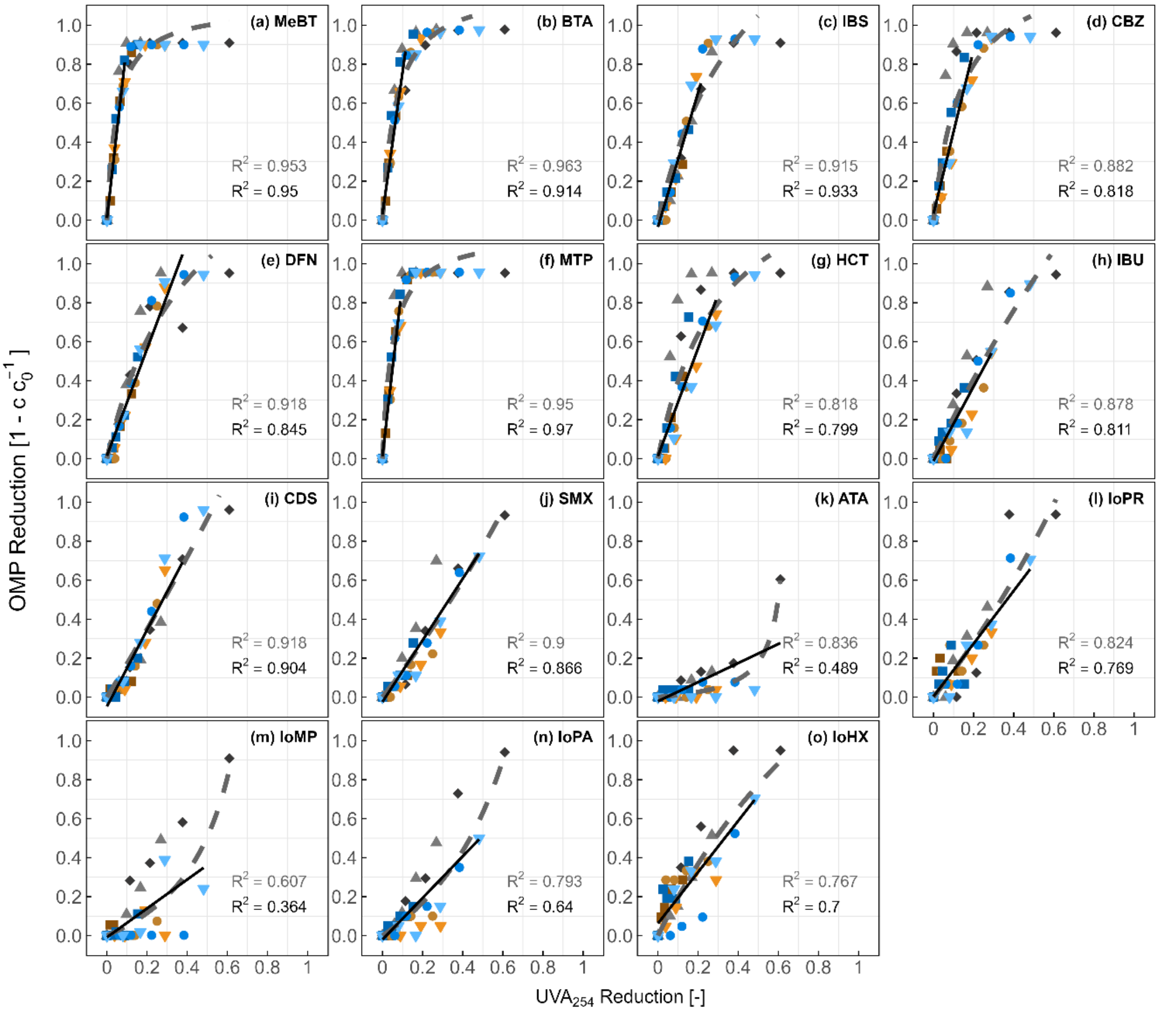

Fig. S6. Correlation between normalized OMP (1 – C C_0^{-1}) and UVA_{254} reduction. Linear correlation (solid black line) was calculated for OMP values <0.8 and corresponding UVA_{254} values. Non-linear correlation (grey dashed line) was calculated using the Equation 2 ($OMP_{Reduction} = (a\ UVA_{254,Reduction})/(1 + b\ UVA_{254,Reduction})$).

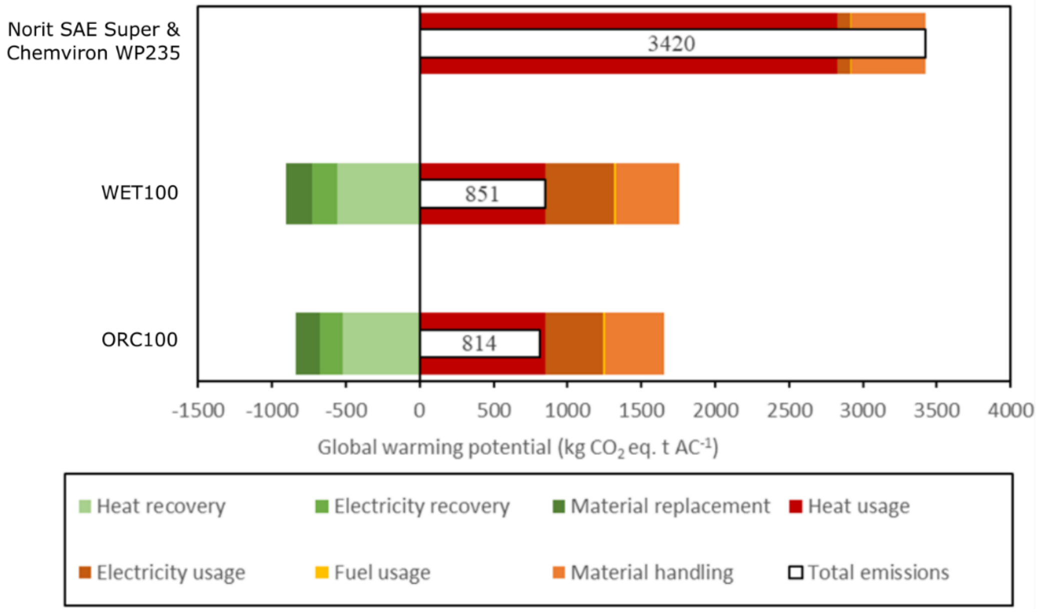

Fig. S7. Global warming potential (GWP) generated during the production of activated carbon derived from landscape conservation material (WET100 and ORC100) and Conventional coal (Norit SAE Super and Chemviron WP235). Each section of the horizontal bars indicates the amount of GWP emitted or saved by the respective parameters as indicated in the legend. The negative values indicate GWP savings, and the positive values indicate GWP emissions, therefore a lower value would mean the option has a lower GWP.

Table S5. Zeta potential and full-width half-maximum (FHWM) of activated carbon.

Activated Carbon	pH	Zeta potential [mV]	FWHM [mV]
ORC0	8.03	-16.7	13.5
ORC50	8.09	-11.5	12.0
ORC100	8.23	-12.7	12.5
WET0	8.20	-15.9	12.0
WET50	8.09	-11.5	11.0
WET100	8.14	-12.0	10.6
Norit SAE Super	8.14	-12.4	17.5
WP 235	8.01	-17.7	20.5

References

Boehler M, Zwickenpflug B, Hollender J, Ternes T, Joss A, Siegrist H. Removal of micropollutants in municipal wastewater treatment plants by powder-activated carbon. Water science and technology a journal of the International Association on Water Pollution Research 2012;66(10):2115–21. https://doi.org10.2166/wst.2012.353.

Eidgenössische Anstalt für Wasserversorgung, Abwasserreinigung und Gewässerschutz. Einsatz von Pulveraktivkohle zur Elimination von Mikroverunreinigungen aus kommunalem Abwasser. Duebendorf; 2010.
